# USP25 Elevates SHLD2‐Mediated DNA Double‐Strand Break Repair and Regulates Chemoresponse in Cancer

**DOI:** 10.1002/advs.202403485

**Published:** 2024-05-27

**Authors:** Yunhui Li, Lei Li, Xinshu Wang, Fei Zhao, Yuntong Yang, Yujuan Zhou, Jiyuan Zhang, Li Wang, Zeshan Jiang, Yuanyuan Zhang, Yuping Chen, Chenming Wu, Ke Li, Tingting Zhang, Ping Wang, Zhiyong Mao, Weiguo Zhu, Xingzhi Xu, Shikang Liang, Zhenkun Lou, Jian Yuan

**Affiliations:** ^1^ Medical Innovation Center Shanghai East Hospital School of Medicine Tongji University Shanghai 200120 China; ^2^ Cancer Center Tongji University School of Medicine Shanghai 200331 China; ^3^ Department of Biochemistry and Molecular Biology Tongji University School of Medicine Shanghai 200331 China; ^4^ College of Biology Hunan University Changsha 410082 China; ^5^ Department of General Surgery and Colorectal Surgery Shanghai East Hospital Tongji University School of Medicine Shanghai 200120 China; ^6^ Translational Research Institute of Brain and Brain‐Like Intelligence Shanghai Fourth People's Hospital School of Medicine Tongji University Shanghai 200080 China; ^7^ State Key Laboratory of Bioactive Substance and Function of Natural Medicines Institute of Medicinal Biotechnology Chinese Academy of Medical Sciences & Peking Union Medical College Beijing 100050 China; ^8^ Tongji University Cancer Center Shanghai Tenth People's Hospital School of Medicine Shanghai 200072 China; ^9^ Shanghai Key Laboratory of Maternal‐Fetal Medicine Clinical and Translational Research Center of Shanghai First Maternity and Infant Hospital Frontier Science Center for Stem Cell Research Tongji University School of Medicine Shanghai 200040 China; ^10^ International Cancer Center Guangdong Key Laboratory of Genome Instability and Human Disease Prevention Marshall Laboratory of Biomedical Engineering Department of Biochemistry and Molecular Biology Shenzhen University Medical School Shenzhen 518037 China; ^11^ The Sixth Affiliated Hospital of Shenzhen University Guangdong Key Laboratory for Genome Stability and Disease Prevention and Carson International Cancer Center Marshall Laboratory of Biomedical Engineering Shenzhen University School of Medicine Shenzhen 518055 China; ^12^ School of Biomedical Sciences LKS Faculty of Medicine The University of Hong Kong Hong Kong SAR 999077 Hong Kong; ^13^ Department of Oncology Mayo Clinic Rochester MN USA

**Keywords:** cancer therapy, deubiquitinase USP25, DNA repair pathways, peptides, SHLD2

## Abstract

DNA damage plays a significant role in the tumorigenesis and progression of the disease. Abnormal DNA repair affects the therapy and prognosis of cancer. In this study, it is demonstrated that the deubiquitinase USP25 promotes non‐homologous end joining (NHEJ), which in turn contributes to chemoresistance in cancer. It is shown that USP25 deubiquitinates SHLD2 at the K64 site, which enhances its binding with REV7 and promotes NHEJ. Furthermore, USP25 deficiency impairs NHEJ‐mediated DNA repair and reduces class switch recombination (CSR) in *USP25*‐deficient mice. USP25 is overexpressed in a subset of colon cancers. Depletion of USP25 sensitizes colon cancer cells to IR, 5‐Fu, and cisplatin. TRIM25 is also identified, an E3 ligase, as the enzyme responsible for degrading USP25. Downregulation of TRIM25 leads to an increase in USP25 levels, which in turn induces chemoresistance in colon cancer cells. Finally, a peptide that disrupts the USP25‐SHLD2 interaction is successfully identified, impairing NHEJ and increasing sensitivity to chemotherapy in PDX model. Overall, these findings reveal USP25 as a critical effector of SHLD2 in regulating the NHEJ repair pathway and suggest its potential as a therapeutic target for cancer therapy.

## Graphical abstract

USP25 deubiquitinates SHLD2 at the K64 site, which enhances its binding with REV7 and promotes NHEJ pathway. A peptide is identified to disrupt the USP25‐SHLD2 interaction, impair NHEJ pathway, and increase sensitivity to chemotherapy in PDX model, thereby proposing a potential therapeutic strategy for cancer therapy.

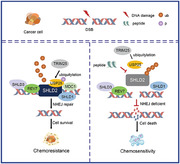

## 1 Introduction

The DNA damage response (DDR) system is critical to maintain genomic integrity and guard against DNA damage. Dysfunction in the DDR pathway results in genomic instability which is a key driving force to the initiation and progression of tumorigenesis. DNA double‐strand breaks (DSBs) are the most lethal lesions, which can trigger a series of cellular DNA damage responses.^[^ ^1^, ^2^ ^]^ DSBs are repaired by two major pathways, namely homologous recombination (HR) and nonhomologous end joining (NHEJ). The NHEJ pathway repairs 75% of DSBs in proliferating cells and occurs throughout the cell cycle.^[^ ^3^ ^]^ Shielding DNA ends is a central process in initiating the NHEJ repair of DSBs. Recent reports have identified the shieldin complex as a new effector of 53BP1‐associated activities that facilitates NHEJ repair and limits DNA end resection.^[^ ^4^, ^5^, ^6^, ^7^ ^]^ The shieldin complex is composed of C20orf196 (SHLD1), FAM35A (SHLD2), CTC‐534A2.2 (SHLD3), and REV7,^[^ ^8^, ^9^ ^]^ with SHLD2 being the key regulator in the complex. The N‐terminal of SHLD2 binds to REV7, facilitating SHLD2 recruitment to DSBs. The C‐terminal of SHLD2 binds to ssDNA ends, preventing the resection of DNA ends by competing with EXO1 and DNA2.^[^ ^10^, ^11^ ^]^ SHLD2 is essential for promoting NHEJ and antibody class switch recombination (CSR).^[^ ^12^ ^]^ High expression of SHLD2 is associated with poor prognosis in breast cancer patients.^[^ ^5^, ^13^ ^]^ However, little is known about the regulatory mechanisms governing SHLD2 activity.

Post‐translational modifications (PTMs) play crucial roles in regulating protein function. Many PTMs of key regulators in the NHEJ pathway have been shown to be associated with tumorigenesis and cancer therapy. Specifically, phosphorylation of the S/T‐Q sites at the N‐terminus of 53BP1 by ATM is required for DNA end protection and immunoglobulin class switch recombination (CSR),^[^ ^14^, ^15^ ^]^ promoting its interaction with RIF1, activating NHEJ repair and cancer cell survival.^[^ ^16^, ^17^ ^]^ Furthermore, 53BP1 ubiquitylation on K1268 by RNF168 is critical for NHEJ repair, maintaining genomic stability and radiosensitivity.^[^ ^18^, ^19^, ^20^ ^]^ However, the PTMs regulating SHLD2 have not been extensively characterized.

In this study, we demonstrate that the deubiquitinase USP25 interacts with SHLD2 and removes Lys63‐linked polyubiquitin chains from SHLD2, facilitating its binding with REV7 and promoting NHEJ. Following DNA damage, ATM phosphorylates USP25 at Thr523, enhancing its binding to MDC1 and recruitment to DSBs. Importantly, we find that USP25 is overexpressed in a subset of colon cancers. Depletion of USP25 sensitizes colon cancer cells to IR, 5‐Fu, and cisplatin. We design a cell‐penetrating peptide that disrupts the USP25‐SHLD2 interaction, which in turn impairs NHEJ repair and ultimately sensitizes cancer cells to chemotherapy in the patient‐derived xenografts (PDX) model. These results reveal a novel regulatory mechanism for the NHEJ pathway and suggest a potential therapeutic strategy based on targeting the USP25‐SHLD2 axis in cancer cases with hyperactivated NHEJ.

## 2 Results

### 2.1 USP25 Interacts with and Deubiquitinates SHLD2

**Figure 1:**
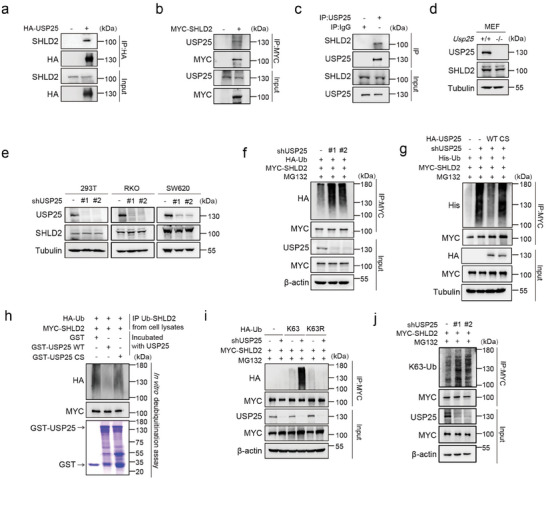
USP25 interacts with and deubiquitinates SHLD2. a) Co‐immunoprecipitation (Co‐IP) analysis was performed to investigate the interaction between USP25 and SHLD2 in HEK293T cells. Cell lysates were subjected to HA resin and subsequently immunoblotted with indicated antibodies to detect the precipitated proteins. b) Co‐IP analysis was additionally performed to examine the interaction between SHLD2 and USP25 in HEK293T cells. Cell lysates were subjected to MYC resin, and the immunoprecipitations were then probed using the indicated antibodies. c) Co‐IP assay of the interaction between USP25 and SHLD2 using antibodies to USP25 in HEK293T cells. Lysates from cells were prepared for co‐IP experiments with USP25 antibody and then blotted with the indicated antibodies. d) The SHLD2 protein levels in *Usp25* deleted mouse embryonic fibroblasts (MEFs) or e) USP25 knockdown HEK293T, RKO, SW620 cells were assessed by immunoblotting. The cells were lysed and subjected to Western blot analysis using the indicated antibodies. f) Control and USP25 knockdown cells were treated with MG132 for 4 h prior to harvest. MYC was immunoprecipitated and blots were then probed with the indicated antibodies. g) Control cells and USP25 knockdown cells reconstituted with USP25^WT^ and USP25^C178S^ mutant were treated to MG132 for 4 h before harvest. MYC was immunoprecipitated and blots were then probed with the indicated antibodies. h) Deubiquitination of SHLD2 in vitro by USP25. Ubiquitinated MYC‐SHLD2 was incubated with purified USP25^WT^ and USP25^C178S^ mutant in vitro and then probed with the indicated antibodies. i) HA‐Ublysine‐specific mutant constructs were transfected into control or USP25 knockdown cells and cells were treated to MG132 for 4 h before harvest. Blots were probed with the indicated antibodies. j) Control and USP25 knockdown cells were treated with MG132 for 4 h prior to harvest. MYC was immunoprecipitated and blots were then probed with the indicated antibody.

To identify potential deubiquitinases (DUBs) that can deubiquitinate SHLD2, we first screened a set of DUBs through coimmunoprecipitation (co‐IP) in order to determine which DUBs interact with SHLD2 (Figure S1a, Supporting Information). Our results identified that USP25 interacted with SHLD2, which was confirmed through co‐IP experiments in HEK293T and RKO cells (**Figure** **1**a–c; Figure S1b, Supporting Information). We further investigated whether USP25 regulated SHLD2 protein levels, however, depletion of USP25 didn't affect SHLD2 protein levels (Figure 1d,e).

Given that USP25 is a key deubiquitinase, we sought to investigate whether USP25 deubiquitinates SHLD2. Our results showed that USP25 depletion led to the promotion of SHLD2 ubiquitination (Figure 1f; Figure S1c, Supporting Information). Rescuing USP25 knockdown cells with USP25^WT^ resulted in decreased SHLD2 ubiquitination, whereas using the USP25^C178S^ mutant, which lacks USP25 deubiquitinating enzyme activity, had no significant effect (Figure 1g). We further confirmed that USP25 directly deubiquitinates SHLD2 in vitro deubiquitination assay. Specifically, purified GST‐USP25^WT^, but not the GST‐USP25^C178S^ mutant, significantly deubiquitinated SHLD2 in vitro (Figure 1h). Additionally, USP25 knockdown increased SHLD2 ubiquitination in cells overexpressing HA‐Ub K63 but not HA‐Ub K63R mutant (Figure 1i; Figure S1d, Supporting Information). We also examined the USP25‐mediated deubiquitination of SHLD2 with the K63‐linkage Ub‐specific antibody and the result indicated that USP25 depletion increased the K63‐linked ubiquitin chain of SHLD2 (Figure 1j). Our findings therefore establish that deubiquitinase USP25 interacts with SHLD2 and catalyzes the K63‐linked polyubiquitination of SHLD2.

### 2.2 USP25 Deubiquitinates SHLD2 to Regulate NHEJ

**Figure 2:**
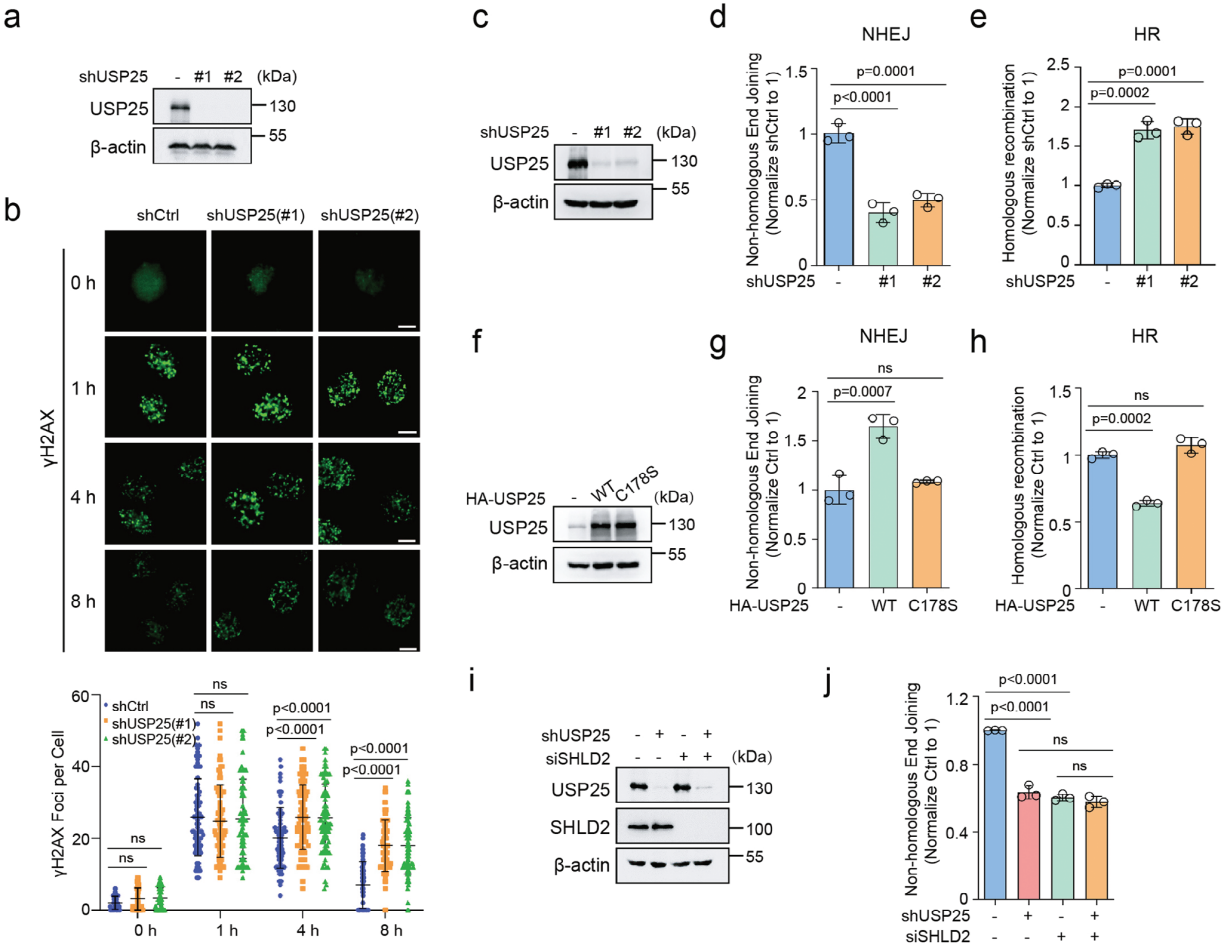
USP25 deubiquitinates SHLD2 to regulate DNA damage repair. a)Immunoblotting was performed to detect USP25 expression in U2OS cells stably expressing either control or USP25 shRNA. b) U2OS cells stably expressing control and USP25 shRNA were left irradiated with or without 2 Gy and probed with γH2AX foci at the indicated time point. Representative micrographs and the quantification of γH2AX foci were shown. n > 80 in each group. c) Immunoblotting was performed to detect USP25 expression in HEK293T cells stably expressing either control or USP25 shRNA. d) NHEJ or e) HR repair capacity of control and USP25 knockdown cells were subjected using a reporter assay. f) Immunoblot of USP25 in HEK293T cells stably expressing control, HA‐USP25^WT^ or HA‐USP25^C178S^ mutant. g) NHEJ or h) HR repair capacity of control, HA‐USP25^WT^, and HA‐USP25^C178S^ mutant cells were assessed using a reporter assay. i) Control and USP25 knockdown HEK293T cells transfected short interfering RNAs (siRNA) against SHLD2 were blotted with the indicated antibodies and j) were then subjected to NHEJ assay. Statistical analysis was performed using two‐way ANOVA followed by a Turkey's multiple comparison test (b) or one‐way ANOVA followed by a Turkey's multiple comparison test (d,e,g,h,j).

Given that SHLD2 plays a key role in promoting NHEJ‐mediated DNA repair, we sought to investigate whether USP25 deubiquitinates SHLD2 to regulate DNA repair. We assessed the formation of γH2AX foci in control and USP25 knockdown cells following IR treatment. Our results showed that knockdown of USP25 in U2OS cells caused more γH2AX foci to sustain at later time points (8 h), indicating that USP25 loss may impair DNA repair (**Figure** **2**a,b). Furthermore, we found that knockdown of USP25 led to decreased NHEJ and increased HR in HEK293T and RKO cells (Figure 2c–e; Figure S1e–g, Supporting Information), and USP25 inhibition by a small molecule inhibitor AZ1 led to similar results as depletion of USP25 in cells (Figure S1h,i, Supporting Information). On the other hand, overexpressing USP25^WT^ but not the USP25^C178S^ mutant increased NHEJ and decreased HR (Figure 2f–h). All these results suggested that USP25 regulated DSBs repair choice. In addition, we observed that knockdown of USP25 decreased NHEJ and increased HR only in WT cells but not in SHLD2‐depleted cells (Figure 2i,j; Figure S1k–m, Supporting Information), suggesting that USP25 regulates DNA repair in a SHLD2‐dependent manner.

To further confirm the role of USP25 in DSBs repair choice, we examined the DNA end resection. Depletion of USP25 promoted RPA32 phosphorylation of Ser4/Ser8 after etoposide treatment (Figure S2a, Supporting Information). Furthermore, RPA32 and RAD51 foci increased in USP25 knockdown cells following IR treatment (Figure S2b,c, Supporting Information). Moreover, we used the restriction enzyme *Asi*SI to generate a double‐strand break in U2OS cell chromosome 1. We then quantified the amplified single‐strand DNA generated from the resulting resection through quantitative PCR (qPCR). Our data showed that more DNA end resection was detected in USP25 knockdown cells compared to the control cells (Figure S2d,e, Supporting Information). Taken together, these findings provide evidence that USP25 plays a critical role in regulating DNA repair.

Previous studies have shown that depletion or inhibition of DNA repair factors led cells to sensitize to DNA damage reagent treatment. We next examined whether USP25 regulates cell survival in response to DNA damage. As shown in Figure S2f–h (Supporting Information), the knockdown of USP25 sensitized cells to IR, 5‐Fu, and cisplatin treatment, while in SHLD2‐depleted cells, the knockdown of USP25 didn't further lead cells to sensitize to these treatments.

### 2.3 K64 as the Major Deubiquitination Site of SHLD2 for NHEJ

**Figure 3:**
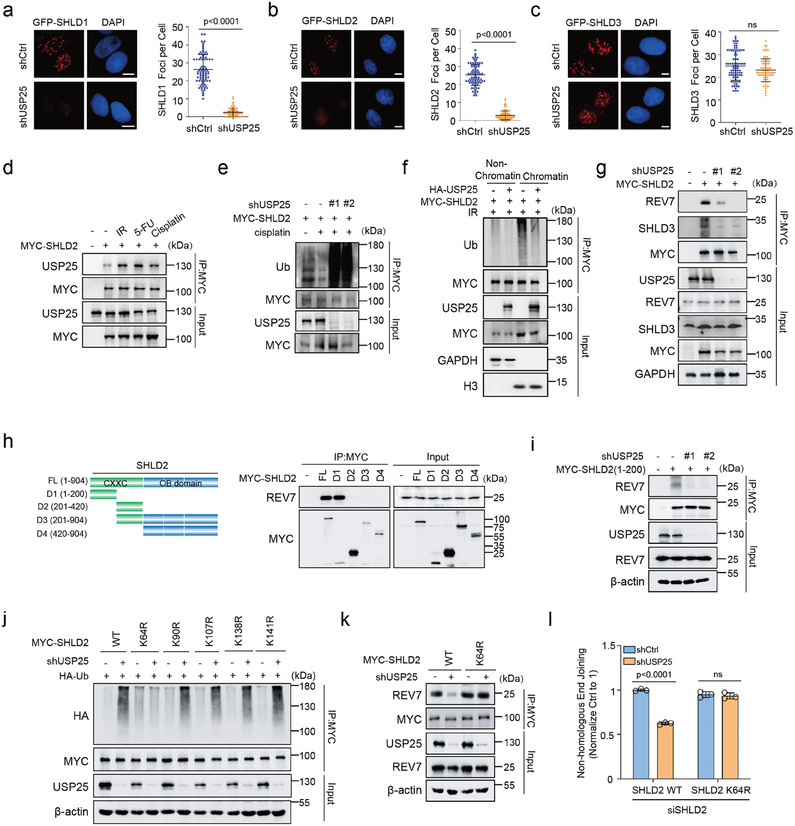
K64 as the major deubiquitination site of SHLD2 for NHEJ.a‐c) U2OS cells stably expressing control or USP25 shRNA were irradiated with 5 Gy and probed with GFP‐SHLD1, GFP‐SHLD2, and GFP‐SHLD3 foci. Cells were stained with anti‐GFP antibodies. Representative micrographs and the quantification of a) GFP‐SHLD1, b) GFP‐SHLD2, and c) GFP‐SHLD3 foci were shown. *n*>80 in each group. d) Myc‐SHLD2 were transfected into RKO cells and cells were left untreated or treated with IR (10 Gy), 5‐Fu (20 um), or cisplatin (20 um), and cell lysates were incubated with the indicated antibody. e) USP25 knockdown RKO cells were transfected with MYC‐SHLD2 and left untreated or treated with cisplatin(20 um). MYC was immunoprecipitated, and blots were probed with the indicated antibodies. f) Cells were treated with IR and added MG132 for 4 h prior to harvest. MYC was immunoprecipitated in chromatin fraction samples or nonchromatin fraction samples. Blots were then probed with the indicated antibodies. g) Myc‐SHLD2 were transfected into control or USP25 knockdown cells. MYC was immunoprecipitated. Blots were probed with the indicated antibodies. h) Schematic representation of MYC‐SHLD2 full length or truncated MYC‐SHLD2. HEK293T cells were transfected with these deletion mutants subjected to anti‐MYC‐affinity gel, and blots were probed with indicated antibodies. i) MYC‐SHLD2 (aa1‐200) were transfected in control or USP25 knockdown cells. MYC was immunoprecipitated. Blots were probed with the indicated antibodies. j) SHLD2 (WT or KR mutants) constructs were transfected into control or USP25 knockdown cells. Myc‐SHLD2 was immunoprecipitated. Blots were probed with the indicated antibodies. k) Myc‐SHLD2 (WT or K64R) constructs were transfected into control or USP25 knockdown cells. Myc was immunoprecipitated. Blots were probed with the indicated antibodies. l) Control or USP25 knockdown HEK293T cells transfected short interfering RNAs (siRNA) against SHLD2 together with wild‐type or K64R Myc‐SHLD2 were subjected to NHEJ assay. Statistical analysis was performed using a *t*‐test (a–c) or one‐way ANOVA followed by Turkey's multiple comparison test (l).

Since USP25 deubiquitinates SHLD2, we further investigate how USP25 regulates the shieldin complex in DNA damage repair. We conducted experiments to examine foci formation of SHLD1, SHLD2, and SHLD3 in USP25 knockdown cells exposed to IR. Our results showed that SHLD1 and SHLD2 foci formation decreased in USP25 knockdown cells, while USP25 depletion did not affect SHLD3 foci, which functions as the upstream factor of SHLD2 (**Figure** **3**a–c). Furthermore, there were no significant changes in 53BP1 and BRCA1 foci formation in USP25 knockdown cells (Figure S3a,b, Supporting Information). Interestingly, the interaction of USP25 with the SHLD2 was increased after DNA damage (Figure 3d) and decreased SHLD2 ubiquitination following DNA damage (Figure 3e). In addition, the USP25‐mediated deubiquitination of SHLD2 was majorly detected in chromatin fraction samples after IR treatment (Figure 3f).

Since SHLD2 recruitment to DSBs is dependent on its binding to REV7 and SHLD3, and depletion of USP25 affects foci formation of SHLD2 but not SHLD3, we hypothesize that USP25 may regulate shieldin complex interaction through deubiquitinating SHLD2. As shown in Figure 3g, USP25 knockdown decreased the binding between SHLD2 and REV7 or SHLD3. Furthermore, we constructed a serial of deletion mutants of SHLD2 and found that the absence of the N‐terminal (aa1‐200) of SHLD2 completely abrogated its binding to REV7 (Figure 3h). Consistent with these findings, USP25 knockdown also impaired the binding between N‐terminal of SHLD2 and REV7 (Figure 3i). Given that N‐terminal of SHLD2 binds to REV7/SHLD3 to facilitate SHLD2 recruitment to DSBs,^[^ ^6^ ^]^ we speculated that USP25 plays a role in regulating shieldin complex formation through deubiquitinating SHLD2 on its N‐terminal region. To examine potential ubiquitination sites on the N‐terminal (aa1‐200) of SHLD2, we analyzed the public PTM proteomic database (https://www.phosphosite.org/). Our analysis showed that there are five potential ubiquitination sites, namely K64, K90, K107, K138, and K141 on the N‐terminal region of SHLD2. We then generated single mutant at these sites and found that K64R abolished the USP25 knockdown‐induced SHLD2 ubiquitination (Figure 3j), suggesting that K64 of SHLD2 is the major deubiquitination site targeted by USP25. In addition, we found that knockdown of USP25 decreased the binding between SHLD2 and REV7, but the SHLD2^K64R^ mutant rescued this phenotype (Figure 3k). Furthermore, the knockdown of USP25 resulted in decreased NHEJ in cells expressing SHLD2^WT^ but not SHLD2^K64R^ (Figure 3l). These results suggest that USP25 deubiquitinates the K64 site on SHLD2, facilitating its binding to REV7 and thereby regulating NHEJ.

### 2.4 Regulation of USP25 by DNA Damage Response Signaling

**Figure 4:**
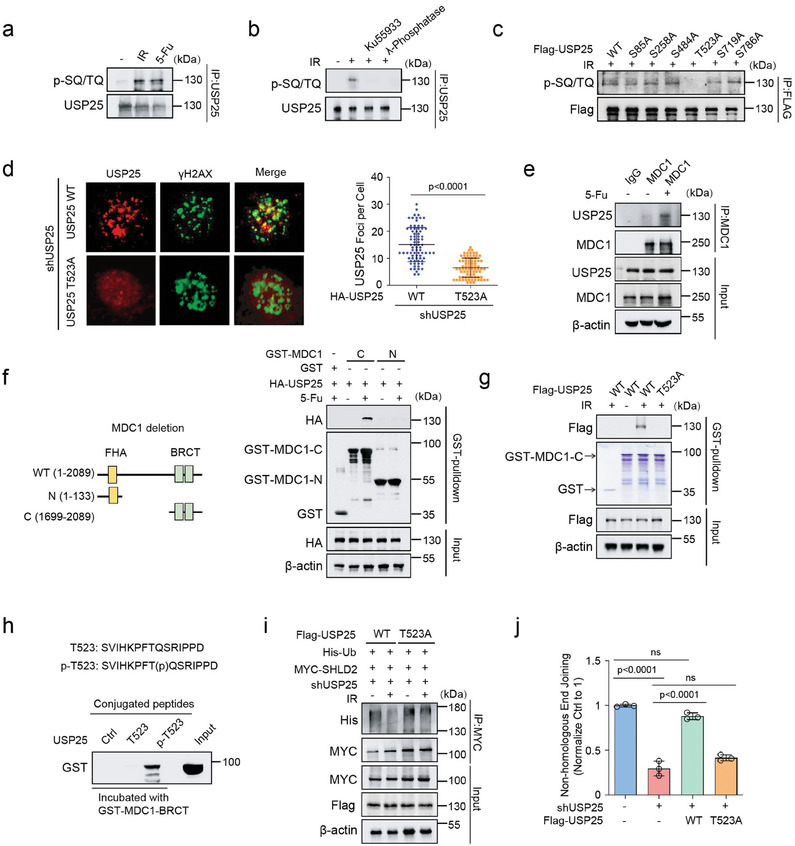
Regulation of the DDR signaling by USP25. a) HEK293T cells were left untreated or treated with IR (10 Gy) or 5‐Fu (20 um). USP25 was immunoprecipitated and immunoblotted with phospho‐SQ/TQ (p‐SQ/TQ). b) HEK293T cells were pretreated with Ku55933 (25 µm) for 2 h followed by treatment with IR(10 Gy). After 1 h, USP25 was immunoprecipitated, left untreated, or treated with phosphatase and immunoblotted with p‐SQ/TQ. c) HEK293T cells transfected with USP25^WT^ and indicated constructs were left treated with IR(10 Gy). USP25 was immunoprecipitated and immunoblotted with p‐SQ/TQ. d) USP25 knockdowm U2OS cells rescued with USP25WT or USP25T523A were treated with IR (2 Gy), and co‐localization USP25 with γH2AX was detected by immunofluorescence. *n*>80 in each group. e) HEK293T cells were left untreated or treated with 5‐Fu (20 um). MDC1 was immunoprecipitated and immunoblotted with USP25. f) HEK293T cells transfected with USP25 were treated with 5‐Fu (20 um), and cell lysates were incubated with GST or GST‐MDC1 deletions in vitro. The interaction USP25 and MDC1 deletions was detected. g) HEK293T cells transfected with USP25^WT^ or USP25^T523A^ were treated with IR, and cell lysates were incubated with GST or GST‐MDC1‐BRCT in vitro. The interaction USP25 and MDC1‐BRCT was detected. h) Non‐phosphorylated or phosphorylated Thr523 peptide was conjugated to beads and incubated with purified GST‐MDC1‐BRCT domain in buffer. Proteins bound to beads were blotted with the indicated antibodies. i) USP25 knockdown cells rescued USP25^WT^ or USP25^T523A^ cells were left untreated or treated with IR(10 Gy). MYC was immunoprecipitated, and blots were probed with the indicated antibodies. j) NHEJ repair capacity of control, USP25 knockdown, and USP25 knockdown rescued USP25^WT^ or USP25^T523A^ cells were subjected using a reporter assay. Statistical analysis was performed using *t*‐test (d) or one‐way ANOVA followed by Turkey's multiple comparison test (j).

Given that USP25 deubiquitinates SHLD2 and facilitates its retention at DSBs, we sought to investigate how USP25 itself is regulated in response to DNA damage. Interestingly, we found that USP25 was phosphorylated following DNA damage at the SQ/TQ motif, which is the consensus site for the ATM/ATR phosphorylation site (**Figure** **4**a). This phosphorylation was abolished by Ku55933 (a specific ATM inhibitor) or λ‐phosphatase treatment, validating that USP25 is phosphorylated by ATM in response to DNA damage (Figure 4b). We then analyzed the USP25 protein sequence and identified six SQ/TQ motifs: S85, S258, S484, T523, S719, and S786. Through generating mutations at each of these sites, we found that the T523A mutation abolished USP25 phosphorylation following IR treatment (Figure 4c). Interestingly, T523 is conserved in human, mouse, rat, zebrafish, monkey, and cattle USP25 sequences (Figure S4a, Supporting Information), suggesting that T523 phosphorylation may play a conserved role in sensing DDR across species.

To better understand the potential role of USP25 phosphorylation in DDR, we stably expressed USP25^WT^ and USP25^T523A^ mutant in USP25 deficient U2OS cells and examined its localization. Our results showed that USP25^WT^ localized to DSBs and co‐localized with γH2AX, while the USP25^T523A^ mutant abolished its DSB localization (Figure 4d). Previous studies have shown that multiple phosphorylated DDR factors bind to the BRCT or FHA domains of mediator proteins, which facilitate them to be loaded to DSBs and form foci.^[^ ^21^ ^]^ We next check whether USP25 phosphorylation facilitates its binding to mediator protein and form foci. We examined several key DDR mediator proteins, including MDC1, 53BP1, and RIF1. Our results revealed that USP25 interacted with MDC1 but not 53BP1 and RIF1 and the USP25‐MDC1 interaction increased following 5‐Fu or IR treatment (Figure 4e; Figure S4b–d, Supporting Information). Furthermore, the MDC1 BRCT‐domain but not FHA‐domain interacted with USP25 following 5‐Fu or IR treatment (Figure 4f; Figure S4e, Supporting Information). Additionally, GST pull‐down assay demonstrated that USP25^T523A^ mutant abolished the binding between USP25 and MDC1 BRCT‐domain, suggesting the importance of T523 phosphorylation for the interaction (Figure 4g). To further confirm the direct interaction between the MDC1 BRCT‐domain and phosphorylated Thr523 of USP25, we incubated T523 or p‐T523 peptide with purified GST‐MDC1 BRCT‐domain in vitro. As shown in Figure 4h, only the p‐T523 peptide bound to the MDC1 BRCT‐domain, indicating a direct binding between the p‐T523 residue of USP25 and the MDC1 BRCT‐domain. We next examined the functional significance of USP25^T523^ phosphorylation. As shown in Figure 4i, DNA damage can only decrease SHLD2 ubiquitination in USP25^WT^ cells but not USP25^T523A^ mutant cells. Moreover, reconstitution of USP25^WT^, but not the USP25^T523A^ mutant, rescued NHEJ repair in USP25 knockdown cells (Figure 4j). These findings support that USP25 phosphorylation by ATM is crucial for its biological function.

### 2.5 USP25 Deficiency Decreases Class Switch Recombination (CSR) in *Usp25* ^−/−^ Mice

**Figure 5:**
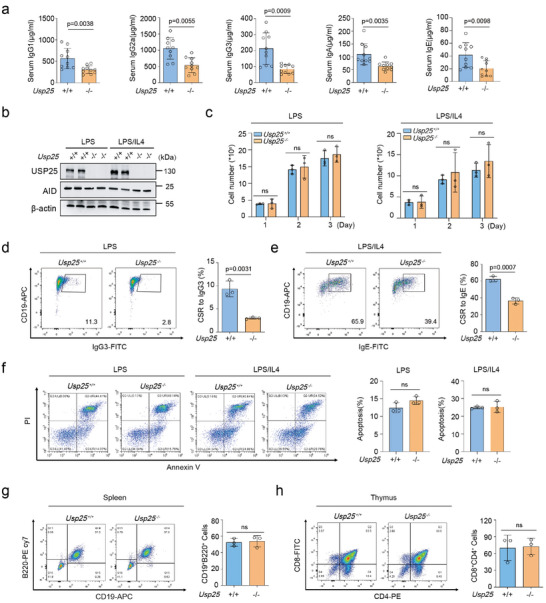
USP25 promotes CSR through NHEJ.a) Serum immunoglobulin was measured in *Usp25^+/+^* and *Usp25^−/−^* mouse. b) B cells isolated from *Usp25^+/+^* and *Usp25^−/−^* mice were treated with LPS or LPS/IL4 for 72 h. Cells were lysed and AID expression levels were plotted for the indicated genotypes. c–e) CSR levels to IgG3 or IgE were measured in *Usp25^+/+^* and *Usp25^−/−^* B cells on day 3 after LPS or LPS/IL4 stimulation. Representative c) growth curve, d,e) flow cytometry blot and quantification of data of indicated B cells. *n*  =  3. f) Apoptotic cell percentages (Annexin V^+^/PI^−^) were assessed on Day 3 after stimulation with LPS or LPS/IL4 and plotted. *n  =*  3. A representative flow cytometry blot and quantitative analysis were presented for the respective B cells. g) CD19^+^ B220^+^ B cell population in spleen of *Usp25^+/+^* and *Usp25^−/−^* mice were shown with flow cytometry and summary graphs. *n  =*  3. h) CD4^+^CD8^+^ T cell population in thymus of *Usp25^+/+^* and *Usp25^−/−^* mice were shown with flow cytometry and summary graphs. *n  =*  3. Statistical analysis was performed using a *t*‐test (a,d,e,f,g,h) or two‐way ANOVA followed by Turkey's multiple comparison test (c).

In line with previous paper, 53BP1, RIF1, and shieldin complex have a well‐documented role in promoting immunoglobulin CSR through the NHEJ pathway.^[^ ^8^, ^11^, ^12^ ^]^ To determine the role of USP25 in immunoglobulin CSR regulation, we generate *Usp25* knockout (KO) mice to study its physiological function. In *Usp25* ^−/−^ mice, serum levels of IgA, IgE, IgG1, IgG2a and IgG3 were reduced compare to *Usp25* ^+/+^ mice (**Figure** **5**a). We isolated native B lymphocytes from the spleens of *Usp25^+/+^* or *Usp25^−/−^* mice, which were then purified and stimulated with lipopolysaccharide (LPS) alone or LPS plus interleukin‐4 (IL‐4) to test the class switch recombination to IgG3 or IgE in vitro. Our results showed that USP25 deficiency did not affect AID protein level or live cell numbers, but class switching for both IgG3 and IgE was decreased in *Usp25^−/−^* B cells after cytokine stimulation (Figure 5b–e). Furthermore, we measured cell apoptosis with Annexin V/PI staining and found no difference between *Usp25^+/+^* and *Usp25^−/−^* spleen B cells after stimulation (Figure 5f). These findings suggest that USP25 may play a critical role in regulating immunoglobulin CSR.

We also examined B220^+^CD19^+^ B cell or CD4^+^CD8^+^ T cell population in spleen or thymus between *Usp25* ^+/+^ and *Usp25^−/−^* mice. There are no differences in lymphocyte composition and differentiation in the spleen or thymus, indicating that impaired CSR in *Usp25* ^−/−^mice is not due to immune system defects (Figure 5g,h). These findings supported that USP25 is essential for immunoglobulin CSR and promotes this process, which is similar to the role of the 53BP1/RIF1/shieldin axis in CSR.

### 2.6 TRIM25 Ubiquitinated USP25 and Antagonized USP25 Function in Cancer Cells

**Figure 6:**
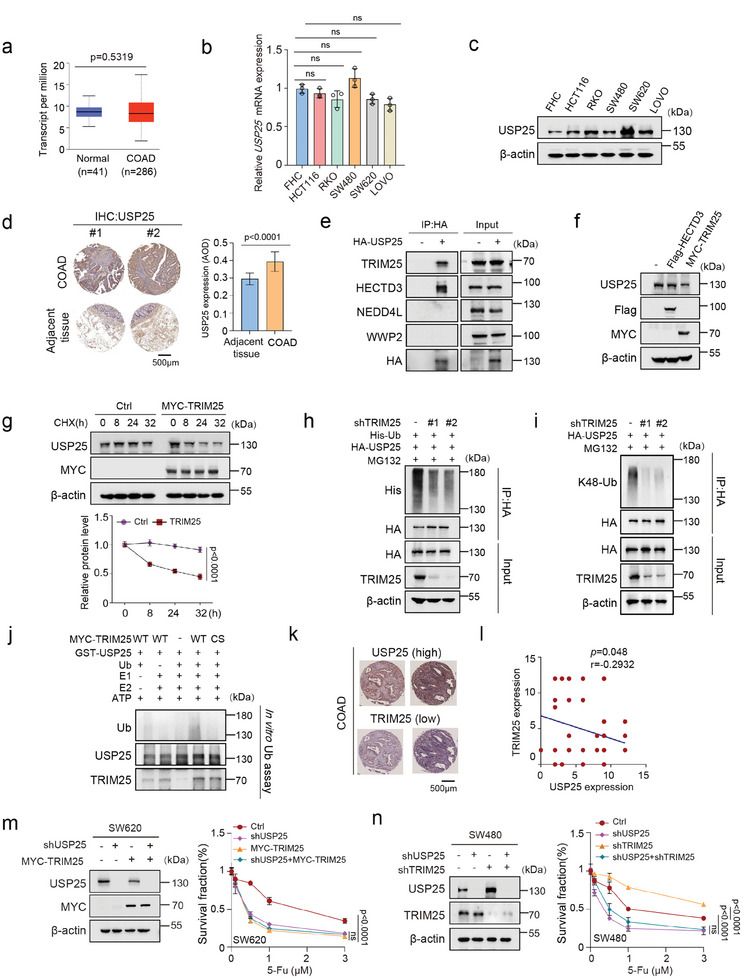
TRIM25 ubiquitinates and inactivates USP25. a) The mRNA expression levels of USP25 in human normal colon tissue samples (*n =* 41) and colorectal adenocarcinoma tissue samples (*n =* 286 COAD patients) from the Ualcan database. b) The mRNA expression level of USP25 in human colon cell lines and colon carcinoma cell lines through qPCR. c) The protein expression level of USP25 in human colon cell lines and colon carcinoma cell lines through western blot. d) Tissue microarray with representative IHC images showing USP25 protein expression in COAD tumors and adjacent tissues. (*n =* 48). e) Co‐IP analysis was performed to detect the interaction between USP25 and TRIM25, HECTD3, NEDDL4, and WWP2. Cell lysates were subjected to HA resin, and the immunoprecipitants were subsequently blotted using the indicated antibodies. f) The protein expression levels of USP25 were evaluated in HEK293T cells transfected with MYC‐TRIM25 or Flag‐HECTD3. g) The effect of cycloheximide (CHX) treatment on USP25 expression levels was analyzed in HEK293T cells transfected with TRIM25 for an indicated duration of time. h) In vivo ubiquitination assays were performed in control and TRIM25 knockdown cells, transfected with indicated plasmids, and treated with MG132 for 4 h before harvesting. Western blot analysis was performed using indicated antibodies. i) Control and USP25 knockdown cells were treated with MG132 for 4 h prior to harvest. HA was immunoprecipitated and blots were then probed with k48‐ub antibodies. j) In vitro ubiquitination assays were conducted by incubating purified GST‐USP25 and MYC‐TRIM25 proteins with recombinant E1, UbcH5a, ubiquitin (Ub), and ATP buffer at 37 °C for 1 h. Samples were immunoblotted using the indicated antibodies. k‐l) Tissue microarray with representative IHC images showing USP25 or TRIM25 protein expression in COAD (*n =* 48 pairs of tumors). Representative images of two different specimens k), and l) the Pearson correlations of staining intensity between USP25 and TRIM25. m) Survival assays for control, USP25 knockdown, MYC‐TRIM25, and USP25 knockdown stably expressing MYC‐TRIM25 SW620 cells for CCK8 assay in response to the indicated concentration of 5‐Fu (0, 0.1, 0.5, 1, 3 µm) for 72 h. n) Survival assays for control, TRIM25 knockdown, USP25 knockdown, and double knockdown SW480 cells for CCK8 assay in response to the indicated concentration of 5‐Fu (0, 0.1, 0.5, 1, 3 µm) for 72 h. Statistical analysis was performed using *t*‐test (d) or one‐way ANOVA followed by Turkey's multiple comparison test or two‐way ANOVA followed by Turkey's multiple comparison test (g,m,n) or Pearson's correlation test (l).

Previous studies have shown that activated DNA repair is response to radio‐chemoresistance in cancers.^[^ ^22^, ^23^ ^]^ We next examined the clinical relevance of USP25 in cancer. Through analysis of the Ualcan dataset and quantitative PCR (qPCR) of colon cancer cell lines, we observed no change in USP25 mRNA levels between colon cancer and normal tissues, the cell lines showed similar results (**Figure** **6**a,b). Interestingly, USP25 protein levels were found higher in several colon cancer cell lines compared to normal cell lines, and a similar phenotype was observed in colon tumor samples (Figure 6c,d). Therefore, we speculated that USP25 protein levels may be regulated by protein post‐translational modification. We searched the GENECARDS database and found a set of E3 ligases that interact with USP25. We then used the Ualcan dataset to select four candidate E3 ligases (TRIM25, HECTD3, NEDD4L, WWP2) whose mRNA levels were lower in colon cancer tissue samples compared to normal colon tissues (Figure S5a, Supporting Information). Co‐IP assays confirmed the interaction between USP25 and TRIM25 or HECTD3, but not NEDD4L and WWP2 (Figure 6e). We further investigated whether TRIM25 or HECTD3 could affect the protein level of USP25 and found that overexpression of TRIM25 but not HECTD3 led to a decrease in USP25 protein level (Figure 6f). Conversely, knockdown of TRIM25 resulted in increased USP25 protein level (Figure S5b, Supporting Information). Furthermore, overexpression of TRIM25 decreased USP25 protein stability (Figure 6g). As expected, the knockdown of TRIM25 notably decreased the polyubiquitination of USP25 (Figure 6h). We also examined the TRIM25‐mediated ubiquitination of USP25 with the K48‐linkage Ub‐specific antibody and found that TRIM25 knockdown decreased the K48‐linked ubiquitin chain of USP25 (Figure 6i). The ubiquitination of USP25 by TRIM25 was further confirmed by in vitro ubiquitination assays using purified GST‐USP25 and MYC‐TRIM25 proteins. Our results showed that TRIM25^WT^ but not the TRIM25^C50S/C53S^ mutant promoted USP25 ubiquitination in vitro (Figure 6j). Additionally, the knockdown of TRIM25 enhanced NHEJ efficiency (Figure S5c, Supporting Information). We also observed a negative correlation between USP25 and TRIM25 protein expression in the colon tumor samples (Figure 6k; Figure S6, Supporting Information). Collectively, these results suggest that TRIM25 promotes USP25 degradation, ultimately acting as a negative regulator of USP25.

We next examined the role of TRIM25‐USP25 axis in radio‐chemoresponse in colon cancers. USP25 was depleted in USP25‐high cell lines (RKO and SW620) and we found that USP25 knockdown sensitized the cells to IR, cisplatin, and 5‐Fu treatment (Figure S5d–i, Supporting Information). Overexpression of TRIM25‐led cells sensitize to 5‐Fu treatment in ctrl SW620 cells (Figure 6m). Conversely, knockdown TRIM25 rendered colon cancer cells resistant to 5‐Fu treatment in ctrl cells but not USP25‐depleted SW480 cells (Figure 6n; Figure S5j–m, Supporting Information). These results demonstrate that TRIM25 regulates colon cancer cell response to 5‐Fu in a USP25‐dependent manner.

### 2.7 USP25‐SHLD2 Axis Confers a Therapeutic Target Against Tumor

**Figure 7:**
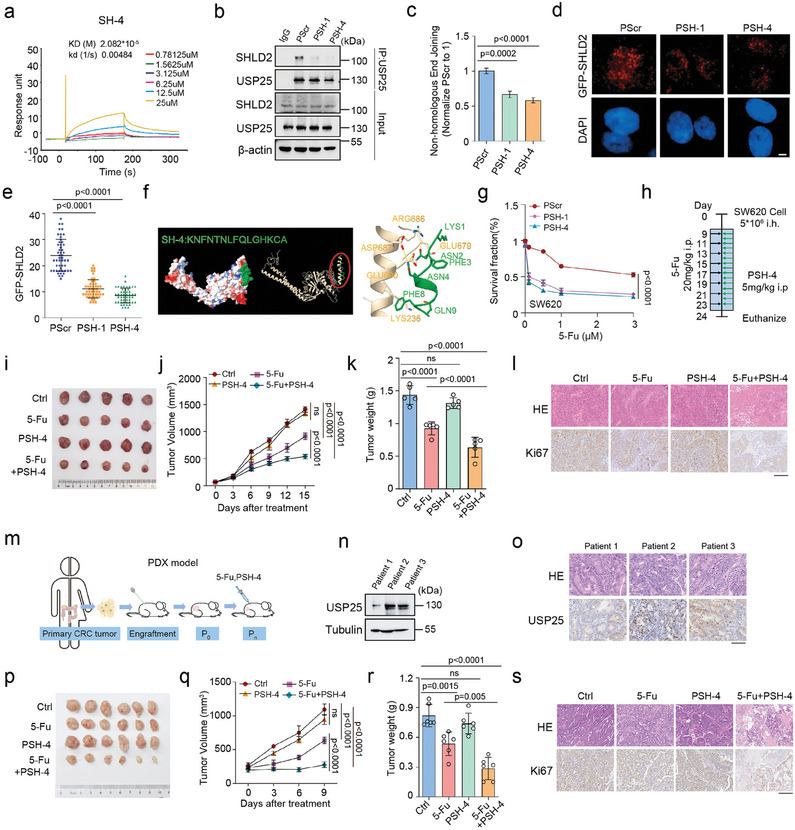
Disturbing the USP25 and SHLD2 interaction counteracts colon cancer progression. a) The kinetic interaction of SH‐4 and USP25 was assessed using surface plasmon resonance (SPR) analyses. b) Co‐immunoprecipitation assays (Co‐IP) were performed to detect the interaction between USP25 and SHLD2 in HEK293T cells treated with indicated peptides. c) NHEJ repair capacity of HEK293T cells treated with indicated peptides was subjected using a reporter assay. d) Representative micrographs of GFP‐SHLD2 foci and e) quantification were shown following irradiation and indicated peptide treatment. Cells were stained with anti‐GFP antibodies. f) The highest‐scoring Dock model of the SH‐4 and USP25 complex is shown. Left: the surface of SH‐4 (green) and USP25 complex. Right: the 3D structure of SH‐4 (green) and USP25 complex. g) The effect of indicated peptides(20 µm) in combination with 5‐Fu (0, 0.1, 0.5, 1, 3 µm) on the chemosensitivity of SW620 cells was determined. h‐l) Xenograft tumor formation by SW620 cells in mice that were untreated or treated with PSH‐4 and 5‐Fu or each treatment alone was analyzed. A h) schematic model, i) representative tumor images, j) tumor volume over time, k) tumor weight, and l) HE or Ki67 staining assay were presented (*n =* 5 animals). m) A schematic model for generating patient‐derived tumor xenografts of colorectal adenocarcinoma (COAD) was provided. n) Western blots showing USP25 levels in COAD patient tumors (patients 1–3) were presented. o) Representative immunohistochemistry (IHC) micrographs showing USP25 expression in COAD patient‐derived tumors were provided. p–s) The PDX xenograft in mice that were untreated or treated with PSH‐4(5 mg k^−1 ^) and 5‐Fu (20 mg k^−1 ^) or each treatment alone were analyzed. Representative p) tumor images, q) tumor volume over time, r) tumor weight, and s) HE or Ki67 staining assay were presented (*n =* 6 animals). Statistical analysis was performed using one‐way ANOVA followed by a Turkey's multiple comparison test (c,e,k,r) or two‐way ANOVA followed by a Turkey's multiple comparison test (g,j,q).

Since activated DNA repair prevents chemosensitivity in cancers, targeting DNA repair pathway may be a potential strategy to increase the susceptibility of cancer cells to the effects of chemotherapy drugs.^[^ ^24^, ^25^ ^]^ Our results showed that knockdown USP25 increased SHLD2 ubiquitination, impaired NHEJ, and led colon cancer cells to sensitivity to chemotherapy. We hypothesized that disrupting USP25‐SHLD2 interaction may downregulate NHEJ and lead colon cancer cells to sensitize to chemotherapy. We screened and designed peptides based on the structure of the N‐terminus (aa 1–200) of SHLD2 using the predictive I‐TASSER server. Surface Plasmon Resonance (SPR) analysis demonstrated that four peptides (SH‐1, SH‐2, SH‐3, and SH‐4) showed the binding affinity for USP25 (**Figure** **7**a; Figure S7a–c, Supporting Information), with SH‐1 and SH‐4 exhibiting the higher affinity. Next, we fused a cell‐penetrating peptide with SH‐1, SH‐4, and a scrambled peptide (Scr),^[^ ^26^ ^]^ generating the chimeric peptides PSH‐1, PSH‐4 and PScr. Upon treatment with these peptides, we found that the PSH‐1 or PSH‐4 disrupted the interaction between USP25 and SHLD2 (Figure 7b). Notably, PSH‐4 showed significantly higher effects in reducing NHEJ and SHLD2 foci formation (Figure 7c–e). Homology modeling based on crystal structure predicted a hydrogen bond between the USP25 protein and the SH‐4 peptide, indicating that the C‐terminus of USP25 is a binding domain of SH‐4 (Figure 7f). We next treated the high USP25 level colon cancer cells SW620 with the peptides and found that the combination of PSH‐1 or PSH‐4 with 5‐Fu killed cancer cells more efficiently (Figure 7g). To further validate the efficacy of PSH‐4 in vivo, we generated xenograft tumor models subcutaneous transplanting with SW620 cells and treatment with PSH‐4 in combination with 5‐Fu. The combination treatment significantly reduced tumor volumes and weights compared to 5‐Fu treatment alone (Figure 7h–k). Ki‐67 positive cells were dramatically decreased in mice combination treated with PSH‐4 and 5‐Fu (Figure 7l).

Furthermore, we evaluated the anti‐tumor effect of PSH‐4 in patient‐derived xenograft (PDX) models (Figure 7m). To assess the levels of USP25 expression, we collected three fresh colon cancer tumors. The western blot and IHC analyses indicated that the tumor from patient 2 exhibited a higher USP25 protein level compared to those from patient 1 and patient 3 (Figure 7n,o). Subsequently, we selected the patient's 2 tumors to establish PDX models and assessed the efficacy of the PSH‐4 and 5‐Fu combination in vivo. The results showed that PSH‐4 synergized with 5‐Fu to reduce tumor volumes and weights in PDX models (Figure 7p–s). Taken together, our findings suggest that the combination of PSH‐4 and chemotherapy reagent may be a potential therapeutic strategy for tumors, especially with high USP25 levels.

## 3 Discussion

DSBs are repaired by two major pathways, namely homologous recombination (HR) and nonhomologous end joining (NHEJ). The NHEJ pathway repairs 75% of DSBs in proliferating cells and occurs throughout the cell cycle.^[^ ^1^, ^2^, ^3^ ^]^ Recently, SHLD2 was identified as a key scaffold protein of the shieldin complex that processes NHEJ repair. The N‐terminal of SHLD2 binds to REV7, facilitating SHLD2 recruitment to DSBs. The C‐terminal of SHLD2 binds to ssDNA ends, preventing the resection of DNA ends and HR repair by competing with EXO1 and DNA2.^[^ ^4^, ^5^, ^6^, ^7^ ^]^ However, the details of SHLD2 regulation following DNA repair remain open questions. In this study, we found that USP25 is involved in the DSB repair pathway by promoting NHEJ through its direct interaction with SHLD2. The interaction of USP25 with the SHLD2 was increased after DSB induction, which in turn decreased SHLD2 ubiquitination following DNA damage. In addition, USP25 catalyzes the K63‐linked polyubiquitination of SHLD2 at K64 and facilitates its binding to REV7, suggesting that ubiquitination of SHLD2 impairs its binding to REV7. Since the SHLD2 ubiquitination K64 site is located at the region bound to REV7, we hypothesized that K63 poly‐ubiquitin chain may change the structure of the binding surface between SHLD2 and REV7. However, further PTM‐based protein complex structures need to be proposed in the future.

Radio‐chemotherapy is the main treatment modality for metastatic or locally advanced cancer, which can induce DNA damage, eventually leading to tumor cell death.^[^ ^27^, ^28^ ^]^ Radiation therapy works by delivering high‐energy radiation to the tumor site, damaging the DNA within cancer cells. These breaks disrupt the normal DNA structure and function, interrupting critical cellular processes and eventually leading to cell death.^[^ ^29^ ^]^ Fluorouracil (5‐Fu) is a chemotherapy drug commonly used to treat colorectal cancer. It belongs to a class of drugs known as antimetabolites. Fluorouracil is metabolized within cancer cells into active compounds that interfere with function of DNA and RNA. It specifically targets rapidly dividing cells, including cancer cells, leading to DNA damage.^[^ ^30^, ^31^ ^]^ Cisplatin is platinum‐based chemotherapy drugs, that bind to the DNA strands and cross–links them, preventing the strands from separating and effectively disrupting DNA replication and transcription processes, evenly leading to DNA damage and triggering cell death pathways in colon cancer cells.^[^ ^32^ ^]^ Hyperactivation of DNA repair may be one of the mechanisms for radio chemoresistance.^[^ ^22^, ^23^ ^]^ Here we found that USP25 may regulate radio‐chemoresponse in a set of colon cell lines. High USP25 levels caused radio‐chemoresistance and USP25 knockdown or inhibition were more sensitive to radio‐chemotreatment.

Previous studies have shown that USP25 plays a negative regulatory role in IL‐17‐triggered signaling and could regulate innate immune response balance.^[^ ^33^ ^]^ However, the composition and number of immune response cells in the spleen and thymus had no difference between *Usp25^+/+^* and *Usp25^−/−^* mice.^[^ ^34^, ^35^ ^]^ Consistent with these findings, our study also indicated that *Usp25* deficiency did not affect the development of B cells and T cells in the spleen or thymus but only affected CSR through the NHEJ deficiency. In addition, several studies have reported the role of USP25 in cancer progression. In previous studies, USP25 was reported to promote Wnt signaling by controlling the levels of tankyrases and deletion of USP25 inhibits the cell growth of colon cancer through tankyrases.^[^ ^36^ ^]^ By screening a compound library, a small molecular, CT1113, was identified as the USP28/USP25 inhibitor, resulting in suppression of the tumor growth in the colon cancer cell line.^[^ ^37^ ^]^ Our results reveal a novel NHEJ regulatory mechanism by USP25 and suggest a potential therapeutic strategy based on targeting the USP25‐SHLD2 axis in cancer cases with hyperactivated NHEJ.

Recently, peptides have been found to have potential for tumor treatment because of their small size, high affinity, easy modification, and low immunogenicity, and utilizing peptides will overcome the limitations of small molecule compounds.^[^ ^38^, ^39^, ^40^ ^]^ Previous paper showed that stable α‐helical peptides as inhibitors of MDM2 and MDMX treated for p53‐dependent cancer.^[^ ^41^, ^42^, ^43^ ^]^ The use of a specific peptide to disrupt the interaction between TRIB3 and MYC in combination with the chemotherapy drug doxorubicin has shown promising results in reducing tumor growth in patient‐derived xenograft models. The peptide may be a potential therapeutic option for treating lymphomas with high TRIB3‐MYC expression.^[^ ^44^ ^]^ Using peptides to target proteins involved in DNA repair pathway may be a new approach to treating tumors. In this study, we found that USP25 is overexpressed in a subset of cancers, leading to activation of the NHEJ pathway and decreased sensitivity to radiotherapy and chemotherapeutic drugs, USP25 may be a good therapeutic target in clinical application. We designed a cell‐penetrating peptide that disrupts the USP25‐SHLD2 interaction, impairing NHEJ repair and sensitizing cancer to chemotherapeutic drugs in the cancer cell lines, CDX, and PDX model. This peptide may be developed as a potential therapeutic option for treating cancer with high USP25 expression.

## 4 Conclusions

**Figure 8:**
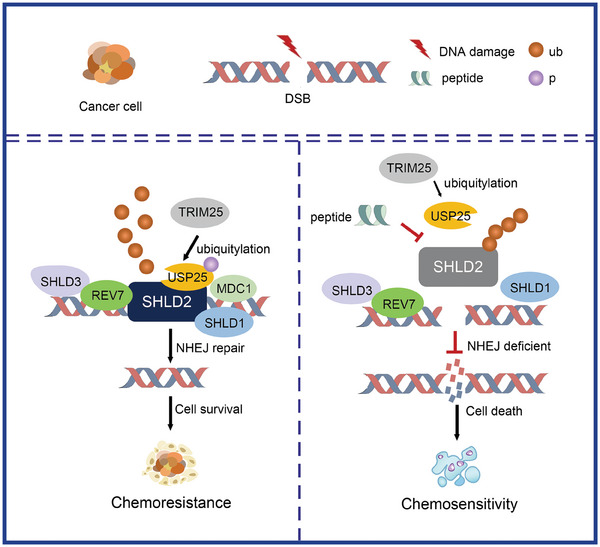
Schematic model. We proposed a model in which USP25 deubiquitinates and modifies SHLD2 by K63‐linked polyubiquitin chains at the K64 site, promoting NHEJ repair. The phosphorylation of USP25 by ATM regulates the ubiquitination of SHLD2, enhances NHEJ repair, and contributes to chemoresistance. Peptide PSH‐4 was identified and found to disrupt the interaction between USP25 and SHLD2. Treatment of PDX mice in combination with PSH‐4 peptide and 5‐Fu significantly increased tumor chemosensitivity.

In conclusion, our study proposes a mechanistic model that USP25 deubiquitinates and modifies SHLD2 with K63‐linked polyubiquitin chains at the K64 site, thereby promoting NHEJ repair, which in turn decreases chemosensitivity in cancer cells. We then developed peptides based on the strategy to disrupt USP25‐SHLD2 binding and demonstrated that the combination of the peptide with chemotherapeutic drug increases killing efficiency in colon cancer cells and PDX model, which implies a potential therapy strategy to increase chemotherapy sensitivity and achieve improved outcomes (refer to **Figure** **8**).

## 5 Experimental Section

### Cell Lines

The cell lines, including HEK293T, U2OS, FHC, RKO, SW480, SW620, HCT116, and LOVO were procured from the American Type Culture Collection. These cell lines were cultured in DMEM and McCoy's 5A medium supplemented with 10% fetal bovine serum (FBS) and penicillin‐streptomycin. The ER‐*Asi*SI U2OS cell line, a kind gift from G. Legube^[^ ^45^ ^]^ was also included in the study and cultured under the same conditions. All cultured cell lines were maintained in a humidified incubator (Thermo Fisher) with 5% CO_2_ at 37 °C.

### Plasmid, shRNAs, and siRNA

USP25^WT^ and USP25^C178S^ mutant genes were cloned into PLVX‐CMV‐HA vector. TRIM25^WT^ and TRIM25^C50S/C53S^ mutant gene was cloned into PLVX‐CMV‐MYC vector. The full‐length and mutant of USP25 gene was cloned into PLVX‐CMV‐Flag vector. The plasmids pCW‐eGFP‐SHLD1, pCW‐eGFP‐SHLD2, and pCW‐eGFP‐SHLD3 were procured from Addgene (#114116, #114119, #114126, #114127). The USP25 and MDC1 expression vectors in *E. coli* were subcloned into the pGEX‐4T‐2 backbone. The full‐length and deletions of SHLD2 were constructed using the PLVX‐CMV‐MYC vector. Site‐directed mutagenesis (Stratagene) was used for creating all site mutants in this study. The shRNAs used in this study were procured from Sigma‐Aldrich, and SHLD2 siRNA was obtained from Dharmacon. The standard method was used for the packaging of lentiviruses for shRNAs. RNAiMax (Invitrogene) was utilized as per the standard protocol for siRNA knockdown. The shRNA sequences and siRNA target sequences utilized in this study were as follows: USP25 shRNA #1: GCTGTAGAAGATATGAGAAAT (TRCN0000004367); USP25 shRNA #2: GCGTGAGCTGAGGTATCTATT (TRCN0000004369); SHLD2 siRNA SMARTpool: siGENOME Human FAM35A siRNA (M‐013761‐01‐0005).

### Antibodies and Drugs

The following drugs were employed: 5‐fluorouracil (5‐Fu: F6627‐1G; Sigma‐Aldrich), cisplatin (S1166, Selleck), cycloheximide (CHX: HY12320, Sigma‐Aldrich), Ku55933 (ab120637, Abcam), λ‐phosphatase (P0753S, New England BioLabs), MG132 (C2211, Sigma‐Aldrich), IPTG (isopropyl‐β‐d‐thiogalactopyranoside, Sigma‐Aldrich, I6758), and 4‐hydroxytamoxifen (4‐OHT; H7904, Sigma‐Aldrich). The concentrations used for each treatment were indicated in the corresponding figure legends.

In the study, the primary antibodies utilized were as follows: anti‐USP25 (Abcam: ab187156, WB:1: 1000; Proteintech: 12199‐1‐AP, IHC: 1: 250; IF: 1: 100; IP: 1: 100); anti‐FAM35A (Novus Biologicals: NBP1‐88980, WB: 1: 500); anti‐C20orf196 (Novus Biologicals: NBP1‐82062, WB: 1: 500); anti‐CTC‐534A2.2 (Novus Biologicals: NBP2‐49564, WB: 1: 1000); anti‐Mad2L2 (Abcam: ab180579, WB: 1: 1000); anti‐γH2AX (Millipore: 05–636, IF: 1: 1000); anti‐phospho‐RPA32(BETHYL: A300‐245A, WB: 1:500); anti‐RPA32 (Santa Cruz: sc‐56770, IF: 1:500); anti‐RAD51 (GENETEX: GTX100469, IF: 1:500); anti‐53BP1 (Novus Biologicals: NB100‐304, WB: 1:1000, IF: 1:500); anti‐BRCA1 (Santa Cruz: sc‐6954, IF: 1:250); anti‐RIF1(Bethyl: A300‐569A, WB: 1:1000); anti‐MDC1 (ABclonal: A8358,WB: 1:500); anti‐p‐SQ/TQ (ABclonal: AP0933, WB: 1:500); anti‐Ub (Santa cruz: sc‐8017, WB: 1:1000); anti‐GST (ABclonal: AE001, WB: 1:1000); anti‐TRIM25 (Proteintech: 12573‐1‐AP, WB: 1:1000, IHC: 1: 250); anti‐WWP2 (Proteintech: 12197‐1‐AP, WB:1:1000); anti‐HECTD3(Proteintech:11487‐1‐AP, WB:1:1000); anti‐NEDD4L (Proteintech:13690‐1‐AP, WB: 1:1000); anti‐Tubulin (Proteintech: 66031‐1‐lg, WB: 1:1000); anti‐GAPDH (ABclonal: A19056, WB: 1:1000); anti‐β‐actin (Sigma: A2228, WB: 1: 2000); anti‐Flag (Proteintech: 20543‐1‐AP, WB: 1:1000); anti‐HA (ABclonal: AE036, WB: 1: 2000); anti‐MYC (Proteintech: 16286‐1‐AP, WB: 1: 1000); anti‐GFP (Abcam: ab290, IF: 1: 1000); anti‐H3 (ABclonal: A2348, IF: 1: 1000); anti‐K48‐Ub (ABclonal: A3606, IF: 1: 1000); anti‐K63‐Ub (CST: 5621S, IF: 1: 500); anti‐AID (ABclonal: A16217, WB: 1:1000); PE‐Cy7 anti‐mouse B220 (BD: 552772, FACS: 1:200); APC anti‐mouse CD19 (BD: 550992, FACS: 1:200); FITC anti‐mouse IgG3 (BD: 553403, FACS: 1:200); FITC anti‐mouse IgE (BD: 553415; FACS: 1:200); FITC anti‐mouse CD8 (BD: 553030; FACS: 1:200); PE anti‐mouse CD4 (BD: 553048; FACS: 1:200).

### Cell Growth Assay

For long‐term proliferation, cells were seeded in triplicate in each well of 96‐well plates at a density of 1000 cells per well. After 24 h of culture, the cells were treated with the indicated doses of regents or peptides and incubated at 37 °C for 72 h. Subsequently, the medium was changed to a fresh medium containing cell counting kit‐8 solution (Dojindo, CK04), and the cells were placed in the incubator for an additional 1–2 h. The absorbance was measured at 450 nm using the SpectraMax M5 reader (Molecular Devices).

### End Resection Detection with qPCR

The protocol for end resection detection using qPCR was previously described.^[^ ^45^ ^]^ In brief, ER‐*Asi*SI U2OS cells were first treated with 4‐hydroxytamoxifen (4‐OHT) for 4 h to induce DSBs. Both control and USP25 knockdown cells were used. After collecting the cells, the genomic DNA was extracted and subsequently digested with BamHI overnight at 37 °C. The primer sequence was described in the previous publication.^[^ ^45^ ^]^ Following digestion, the samples were used as templates in qPCR reactions with Taqman. Three biological replicates were performed for all samples.

### Protein Purification and IN vitro Pull‐Down Assays

*E. coli* BL21 DE3 cells were transformed with human GST‐MDC1 plasmid and induced for 20 h at 20 °C with 1.0 mm IPTG to express GST fusion proteins. The cells were subsequently collected and resuspended in PBS containing 1% Triton X‐100 and 1 mm PMSF, followed by ultrasonication. After centrifugation at 12 000 rpm for 10 min, the supernatant was affinity purified using Glutathione‐Sepharose beads overnight at 4 °C. The purified proteins were then incubated with USP25^WT^ or USP25^T523A^ cell lysates for 2 h at 4 °C, after which the beads were washed with NETN Lysis buffer (0.5% NP40, 2% 1 m Tris HCl, pH 8.0, 2% 5 m NaCl, 0.2% 0.5 m EDTA). The proteins conjugated to the beads were analyzed by western blot.

### In Vivo Deubiquitination and Ubiquitination Assay

For the in vivo deubiquitination assay, USP25 knockdown cells were cultured in a 10 cm dish until they reached a density of ≈50%. The HA‐Ub plasmid was then co‐transfected with MYC‐SHLD2‐WT or MYC‐SHLD2‐KR site mutant plasmids into the stably transfected cells as mentioned above. After transfection for 8 h, the fresh culture medium was replaced. 36 h after transfection, 20 µm of the proteasome inhibitor MG132 was added to the cells, which were then treated for 4 h. The cells were lysed in NETN buffer containing protease inhibitors for 20 min, and cell fragments were subsequently removed by centrifugation. The whole cell extracts were immunoprecipitated with MYC beads, then washed and subjected to immunoblot.

For the in vivo ubiquitination assay, TRIM25 knockdown cells transfected with His‐Ub, HA‐USP25, and treated with proteasome inhibitor MG132 before collecting cells, the remaining steps were same as the deubiquitination experiment in vivo.

### In Vitro Deubiquitination Assay

HEK293T cells were co‐transfected with the MYC‐SHLD2 and HA‐Ub expression plasmids. The Ub‐SHLD2 proteins were purified anti‐MYC‐agarose beads in lysis buffer and eluted with MYC peptide. Recombinant GST‐USP25^WT^ and GST‐USP25^C178S^ were expressed in *E. coli* BL21 DE3 cells and purified according to the standard protocol. Ubiquitinated proteins were incubated with recombinant USP25 in a deubiquitination buffer (50 mm Tris‐HCl pH 8.0, 50 mm NaCl, 1 mm EDTA, 10 mm DTT, 5% glycerol) for 4 h at 30 °C. Input and beads were boiled in a loading buffer and subjected to immunoblot.

### In Vitro Ubiquitination Assay

For the in vitro ubiquitination assay, 50 µL of kinase reaction buffer was prepared, containing: 50 mm Tris, pH 7.4, 1 mm DTT, 2 mm ATP, and 5 mm MgCl_2_. This buffer was then supplemented with 0.5 µg E1 (Boston Biochem), 0.5 µg E2 (UbcH5a; Boston Biochem), and 0.5 µg Ub (Boston Biochem). Purified GST‐USP25 protein and MYC‐TRIM25 WT or CS mutant were incubated in the above reaction buffer for 1 h at 37 °C. Samples were analyzed by immunoblot.

### Mouse Serum Immunoglobulin Detection

Serum immunoglobulins in 10–14‐week‐old mice were assessed using the Mouse Immunoglobulin Isotyping Kit from BioLegend, following the manufacturer's instructions. Briefly, mouse blood samples were centrifuged at 3000 rpm at 4 °C and the serum was appropriately diluted in assay buffer. The diluted serum was then incubated with 25 µL of Ig capture beads at room temperature. Each sample was added 25 µL detection antibody mixture and incubated for 1 h. After two washes with wash buffer, the beads were subjected to analysis using the Flow Cytometer.

### Class Switch Recombination Assay

The mouse splenic B cells were isolated from 10–14 week‐old mice (both male and female) using the EasySep Mouse B Cell Isolation Kit (Stem cell Tech) according to the reagent instructions. Mouse spleen was dispersed in PBS containing 2.5% FBS. Cell aggregation and debris were removed by passing the cell suspension through a 70 µm mesh nylon strainer. The cell suspension was centrifuged for 5 min at 1500 rpm and resuspended in red blood cell lysis buffer. The cells were washed twice with washing buffer after incubation for 10 min at room temperature, adjusting cell density to achieve 1 × 10^8^. Then cells were blocked with rat serum (50 µL mL^−1^).

The isolation cocktail was added to cell suspension (50 µL mL^−1^) and incubated for 12.5 min at room temperature. The RapaidSphere was added to cell suspension (75 µL mL^−1^) and incubated for 2.5 min at room temperature. The tubes were placed into the magnet and incubated for 2.5 min at room temperature. The enriched B cells were cultured in RPMI 1640 supplemented with 10% FBS and 50 µm β‐mercaptoethanol. To induce class switching in mouse B cells, 2 × 10^5^ cells were cultured in a medium supplemented with a mixture of CD19‐FITC‐positive cells were then stained with anti‐IgG3‐PE or anti‐IgE‐PE and the fluorescence signal was analyzed using BD FACS Aria II.

### Peptide Synthesis and Treatment

All peptides were synthesized by SBS Genetech Co., Ltd. (Beijing, China) and were of high purity (>95%), as determined by high‐pressure liquid chromatography. These synthetic peptides were suitable for both in vitro and in vivo applications. Specifically, the SH1, SH2, SH3, and SH4 peptides comprised the following amino acid sequences: MSVADPWKKIQLLYS, EKQHKNLENYKVPESI, FTEEEKYQKLLSENK, and KNFNTNLFQLGHKCA. For in vivo experiments, PScr and PSH4 were dissolved in corn oil and subjected to ultrasound to generate a 20 mm stock solution. For in vitro experiments, PScr, PSH1, and PSH4 were dissolved in PBS and stored at −20 °C.

### The Specimen Microarray, Immunohistochemical Staining, and HE Staining

The COAD specimen microarray was procured from Shanghai Wellbio Biotechnology Co., Ltd. Immunohistochemical staining was performed following the previously described.^[^ ^46^, ^47^ ^]^ Briefly, the slices were dewaxed in xylene, washed with ethanol, and then soaked with PBS for 5 min. Thereafter, they were washed once with PBS containing 0.1% Triton‐100. Antigen retrieval was done by incubating the tissue sections in antigen repair solution, followed by blocking with 5% fetal bovine serum for 2 h. The primary antibody was added to the cassette and incubated overnight at 4 °C, followed by leaving it at room temperature for 20 min. The sections were then washed three times with PBS containing 0.1% Triton 100. The secondary antibody was added and incubated for 2 h at 25 °C. Following the removal of the secondary antibody, the sections were washed three times with PBS containing 0.25% Triton‐100. DAB staining solution was added to each tissue section, followed by stopping the staining with PBS, hematoxylin re‐staining, dehydration, and sealing. Immunostaining was blindly scored by pathologists based on staining intensity and ratio of positive cells. The IHC staining was evaluated and scored based on the staining intensity and proportion of positive cells.

### Tumor Models and COAD PDX

This study obtained all clinical colon cancer samples and was approved by the Ethics Committee of The Shanghai East Hospital of Tongji University (2019tjdx39). 6‐week‐old BALB/C‐Nude mice were employed to create xenograft tumor formations, utilizing a portion of the freshly resected tumor specimens for patient‐derived xenograft (PDX) generation. Multiple fragments obtained from diverse portions of the tumor were subjected to mechanical dissociation to generate 3–4 mm^3^ tissue blocks. Subsequently, the suspensions were immersed in complete media (DMEM, Gibco) and subcutaneously injected using a puncture needle into the left and right flank of the recipient Nude mice to produce a solid tumor xenograft. Recipient mice (P0), when tumor diameter reached 1.5 cm and between 2–3 weeks post‐inoculation, were humanely euthanized, the tumors were removed and re‐implanted into the flanks of mice. For the xenograft assay, SW620 cells were subcutaneously injected in the flanks of six‐week‐old female BALB/C‐Nude mice, using 18‐gauge needles. Each mouse received a subcutaneous injection of a 100 µL mixture containing 5 × 10^6^ cells in PBS and 7:3 with Matrigel (BD Biosciences). Tumor volumes were assessed by multiplying the length × width^2^ × 0.5 utilizing a vernier caliper at the onset of the treatment. Once the mean tumor size reached 150 mm^3^, the mice were randomly and evenly allocated into four intervention groups: the vehicle group, the peptide‐only group PSH‐4(5 mg k^−1 ^ per day) and 5‐Fu group (20 mg k^−1 ^ every two days), and the peptide (5 mg k^−1 ^g per day) combined with 5‐ Fu (20 mg k^−1 ^g every two days) group. The largest tumor volume did not exceed the range permitted by the ethics committee (tumor size ≤2000 mm^3^).

### MEFs Preparation

*Usp25* ^−/−^ mouse embryonic fibroblasts (MEFs) were derived from day‐13.5 embryo pools of *Usp2*5^−/−^ mice by a standard procedure.^[^ ^48^, ^49^ ^]^ In briefly, opened the uterus and dissected the embryos to a culture dish containing PBS. Used forceps and scissors to carefully remove the head and internal organs. Gently scraped and cut to fragment the tissue, obtained embryo fragments. Added 1 mL neutralization solution to digest 25 min and sieved the liquid with a 0.45um sieve. Transferred the liquid to a culture dish containing DMEM and 10% FBS. Added 1% penicillin/streptomycin to prevent bacterial contamination. Placed the culture dish in a cell culture incubator at 37 °C with 5% CO_2_.

### Statistical Analysis

The mean values ± SD of at least three independent experiments were used to generate the bar or line graphs. All findings were considered significant at a *p*‐value threshold of 0.05. Significant *p*‐values were indicated within the figures. The figure legends specified the biological replicates for each experiment. Sample sizes for these experiments were generally determined based on previous studies with similar experiments.^[^ ^50^, ^51^, ^52^ ^]^ No data were excluded from the analyses. The experimental mice were randomly allocated to control and treatment groups from different cages. Immunohistochemistry and immunofluorescence imaging were performed and analyzed in a blinded fashion. However, blinding was not employed for other experiments. The plots and graphs were constructed and analyzed using GraphPad Prism 10.0 and FlowJo X.

### Ethical Statement

The research complied with all relevant ethical regulations. All animal procedures were performed in accordance with protocols approved by Shanghai Model Organisms Center, Inc. (2016‐0027, 2019‐0026, 2022‐0030) and were performed in accordance with guidelines from the Laboratory Animal Care Committee of Shanghai East Hospital of Tongji University.

### Reporting Summary

Further details regarding the research design can be found in the Nature Research Reporting Summary that is linked to this article. https://ualcan.path.uab.edu/tutorial.html. All other data that support the conclusions of this study can be made available at reasonable request to the corresponding author.

## Conflict of Interest

The authors declare no conflict of interest.

## Author Contributions

Y.L., L.L., and X.W. contributed equally to this work. J.Y. and Z.L. conceived and designed the study. Y.L., L.L., X.W., and F.Z. performed experiments, analyzed data, and wrote the manuscript. Y.Z. J.Z. and L.W. carried out xenograft experiments. Z.J. and Y.Y. prepared the figures and edited the manuscript. K.L. and T.Z. conducted the mouse drug toxicology experiment of peptides. J. Y. and Z.L. revised the manuscript. Y.C., C.W., P.W., Z.M., X.X., W. Z., and S.L. provided the constructional suggestions and help for this paper. All authors have read and agreed to the published version of the manuscript.

## Supporting information

Supporting Information

## Data Availability

Data sharing is not applicable to this article as no new data were created or analyzed in this study.
