# Supplementary material for: USP25 Elevates SHLD2‐Mediated DNA Double‐Strand Break Repair and Regulates Chemoresponse in Cancer
Source: Adv Sci (Weinh). 2024 May 27;11(28):2403485. doi: 10.1002/advs.202403485 (PMC11267380; doi:10.1002/advs.202403485)
Supplement: Supplementary file 1 — Supporting Information [file ADVS-11-2403485-s001.docx]

**
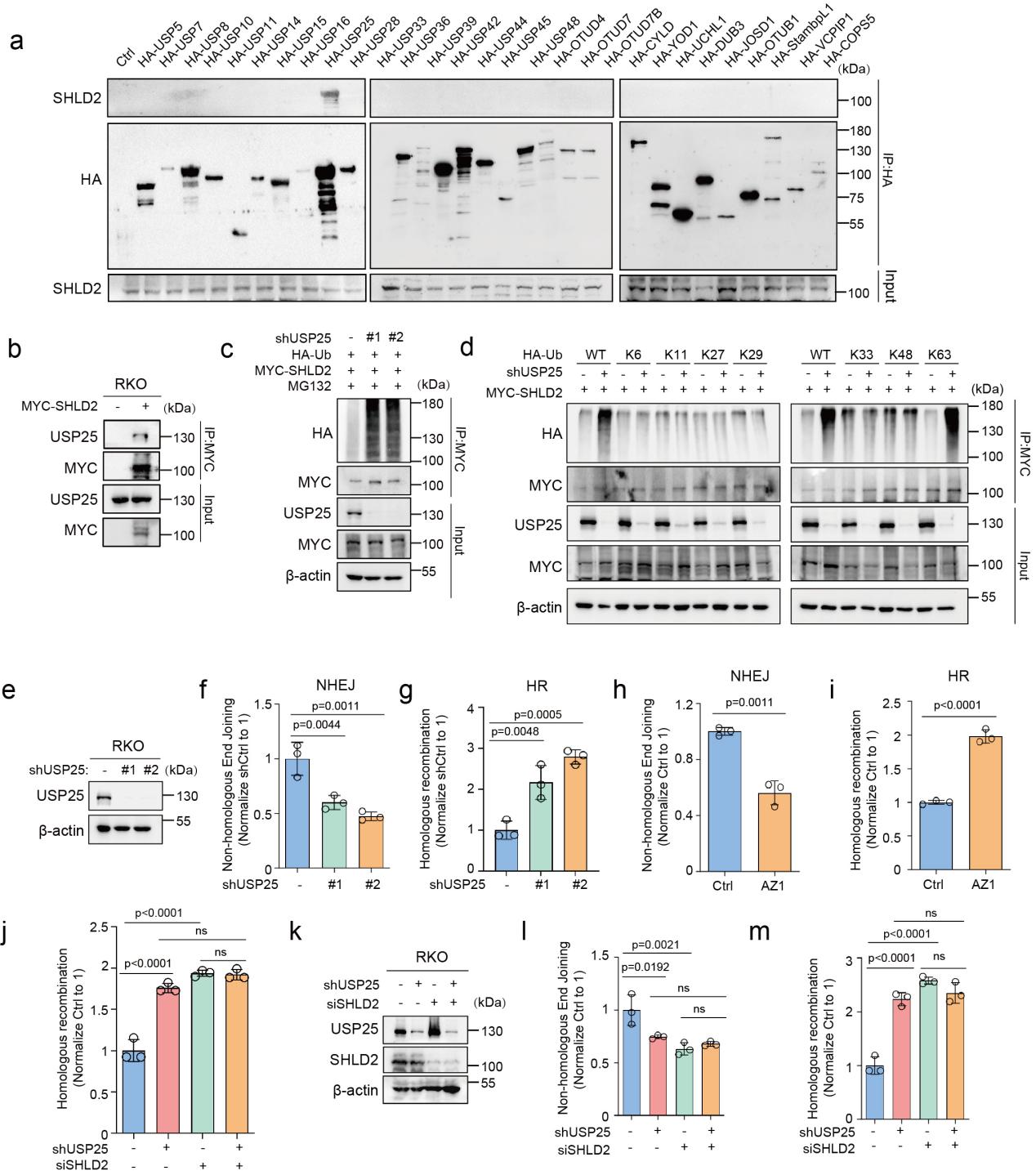
**

**Figure S1. USP25 promotes NHEJ, related to Figure 1-2.**

**a)** A putative SHLD2-interacting deubiquitinase (DUB) screening was performed using a set of different DUBs. Vectors encoding HA-tagged DUBs were transfected into HEK293T cells. Cells were harvested for a co-IP assay. Western blot analysis with the indicated antibodies. **b)** Co-IP analysis was additionally performed to examine the interaction between SHLD2 and USP25 in RKO cells. Cell lysates were subjected to MYC resin, and the immunoprecipitations were then probed using the indicated antibodies. **c)** Control and USP25 knockdown RKO cells were treated with MG132 for 4 hours prior to harvest. MYC was immunoprecipitated and blots were then probed with the indicated antibodies. **d)** Immunoprecipitation analysis of cells expressing Myc-SHLD2 with wild type (WT) HA-Ub or HA-Ub mutants containing a lysine residue only at position 6, 11, 27, 29, 33, 48, or 63 alone. Immunoblotting was performed to detect with the indicated antibodies. **e**) Immunoblotting was performed to detect USP25 expression in RKO cells stably expressing either control or USP25 shRNA. **f-g)** NHEJ (f) or HR (g) repair capacity of control and USP25 knockdown cells were subjected using a reporter assay. **h-i)** NHEJ (h) or HR (i) repair capacity of cells treated with AZ1 were subjected using a reporter assay. **j)** The homologous recombination (HR) assay was conducted in control and USP25 knockdown HEK293T cells transfected with short interfering RNAs (siRNA) against SHLD2. **k-m)** Control and USP25 knockdown RKO cells transfected short interfering RNAs (siRNA) against SHLD2 were blotted with the indicated antibodies (k) and were then subjected to NHEJ (l) or HR (m) assay. Statistical analysis was performed using one-way ANOVA followed by a Turkey’s multiple comparison test (f-j,l-m).

**
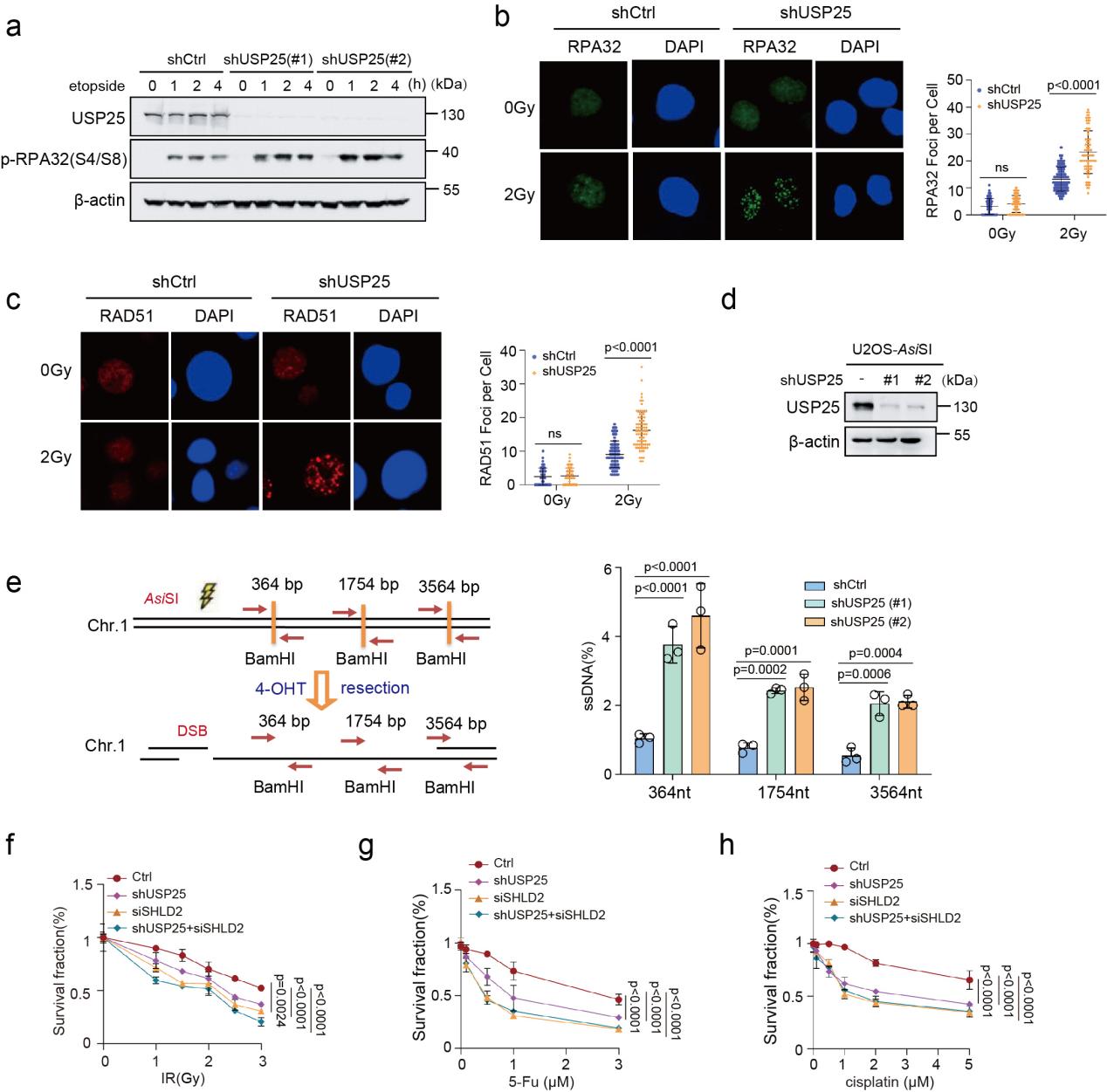
**

**Figure S2. USP25 is involved in DNA damage repair , related to Figure 2.**

**a)** HEK293T cells stably expressing control or USP25 shRNA were untreated and treaded with etopside at the indicated time point. Cells were lysed and lysates was performed with the indicated antibodies. **b-c)** Representative foci images and quantification of RPA32 (b) and RAD51 (c) were obtained in U2OS cells stably expressing control or USP25 shRNA with or without IR treatment (2Gy). n>80 in each group. **d)** USP25 was knocked down in ER-*AsiS*I U2OS cells by shRNA, and cell lysates were analyzed using the indicated antibodies. **e)** Schematic representation of Taqman qPCR primer design for measurement of DSB% at *Asi*SI sites located on chromosome1(Chr.1). The primer pairs of DNA resection are across BamHI restriction sites are marked by red arrows. ER-*AsiS*I U2OS cells stably expressing control and USP25 shRNA were treated with 300 nM 4- hydroxytamoxifen (4-OHT) for 4 h or mock treated and genomic DNA was then extracted and digested with BamHI overnight. The percentages of ssDNA at various distances (364nt, 1754nt and 3564nt) were measured by qPCR. **f-h)** Control and USP25 knockdown RKO cells transfected short interfering RNAs (siRNA) against SHLD2 were subjected to survival assays for CCK8 assay in response to IR (0, 1,1.5,2,2.5,3Gy), 5-Fu (0, 0.1,0.5,1,3µM) or cisplatin(0, 0.1,0.5,1,2,5µM). Statistical analysis was performed using two-way ANOVA followed by a Turkey’s multiple comparison test (b,c,e,f,g,h) .

**
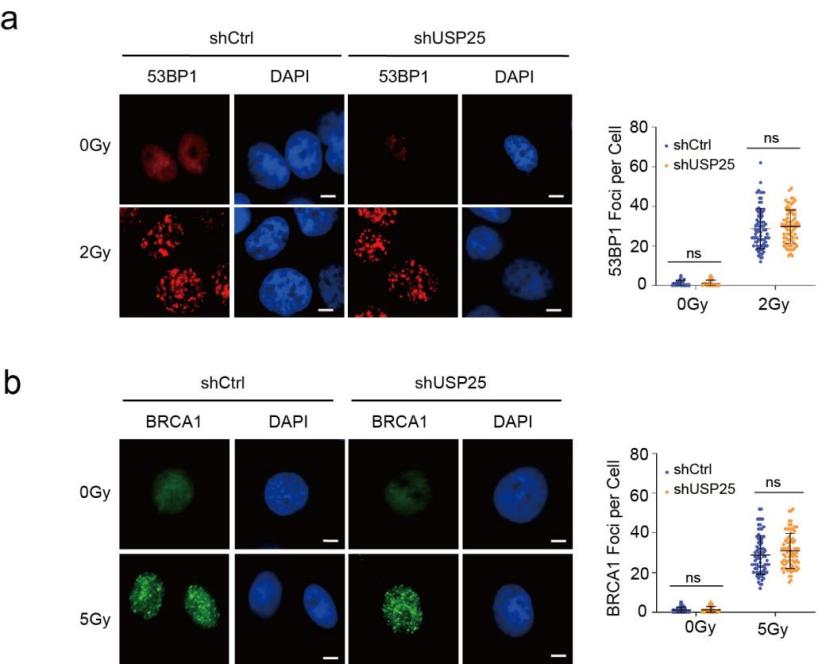
**

**Figure S3. 53BP1 or BRCA1 foci formation in the USP25 knockdown cells, related to Figure 3.**

**a-b)** U2OS cells stably expressing control and USP25 shRNA were treated with or without IR (2Gy or 5Gy) and probed with 53BP1 or BRCA1 foci. Representative micrographs and the quantification of 53BP1(a) or BRCA1(b) foci were shown. n>80 in each group. Statistical analysis was performed using two-way ANOVA followed by a Turkey’s multiple comparison test (a,b) .


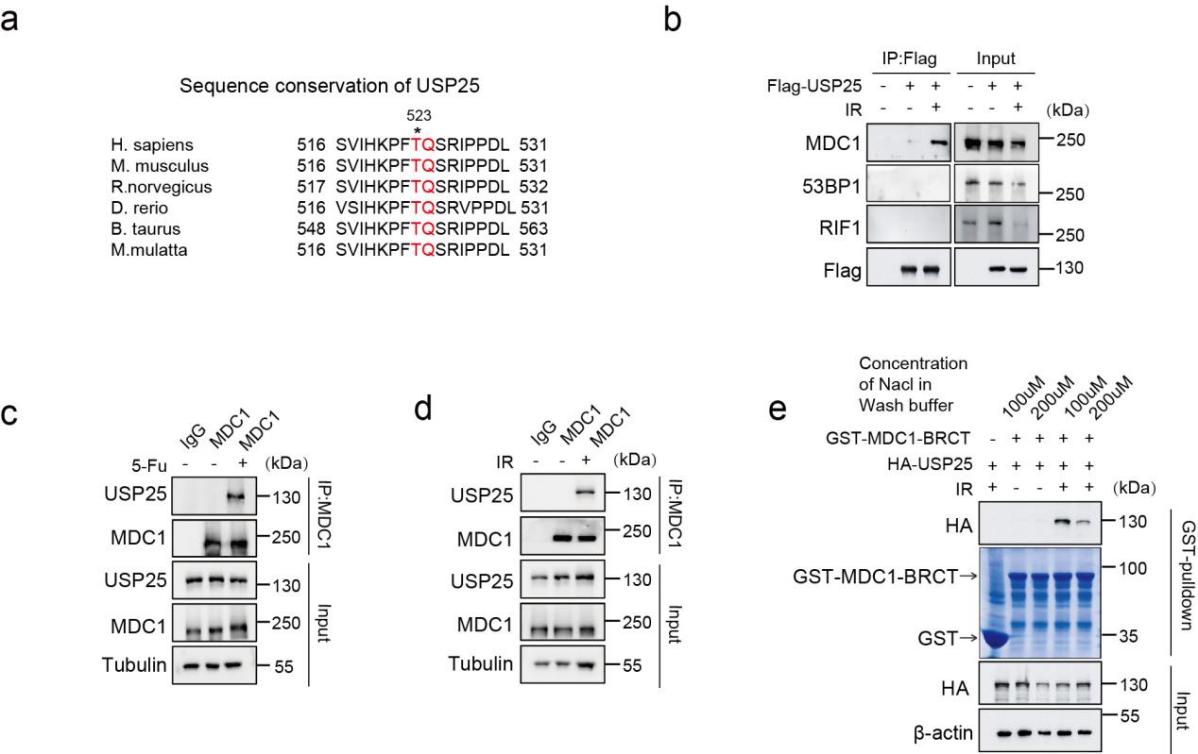


**Figure S4. Regulation of the DDR signaling by USP25, related to Figure 4.**

**a)** The amino acid sequences of USP25^T523^ in human, house mouse, norway rat, zebrafish, cattle, and rhesus monkey are presented. **b)** HEK293T cells overexpressing USP25 were subjected to either no treatment or treatment with IR(10Gy). Flag was immunoprecipitated and immunoblotted with indicated antibodies. **c-d)** RKO cells were left untreated or treated with 5-Fu (20uM)(c) or IR(10Gy)(d). MDC1 was immunoprecipitated and immunoblotted with indicated antibodies. **e)** Purified recombination GST or GST-MDC1-BRCT were incubated with HA-USP25 *in vitro* followed by washing with buffers with different salt concentrations. The interaction USP25 and MDC1-BRCT was detected.

**
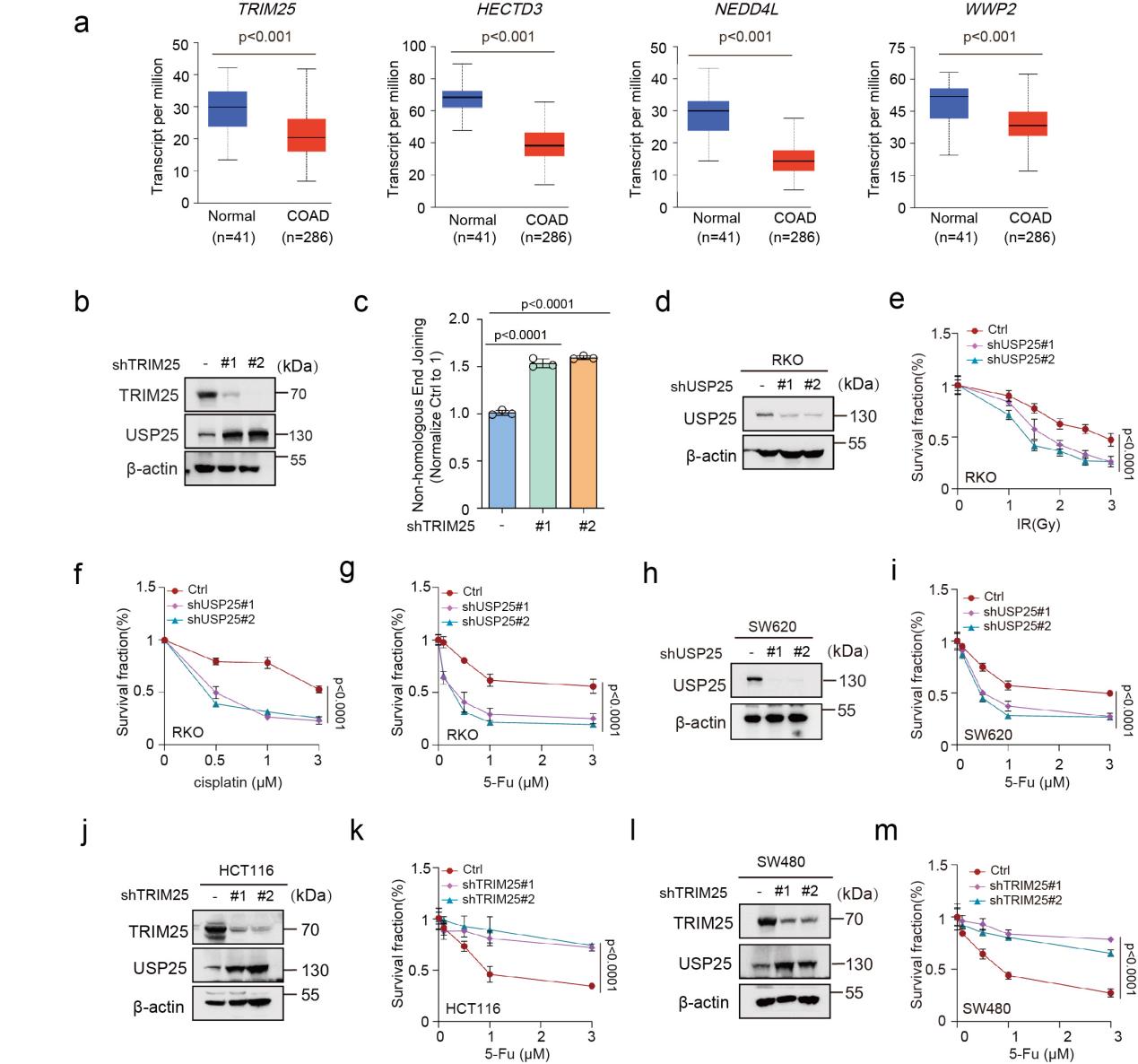
**

**Figure S5. The role of USP25 and TRIM25 in response to chemotherapy drug treatment, related to Figure 6.**

**a)** The mRNA expression levels of *TRIM25*, *HECTD3*, *NEDD4L* and *WWP2* in human normal colon tissue samples (n=41) and colorectal adenocarcinoma tissue samples (n=286 COAD patients) from the Ualcan database were analyzed. **b)** The protein expression levels of USP25 were measured in HEK293T cells that stably expressed TRIM25 shRNA. **c)** NHEJ repair capacity of control or TRIM25 knockdown cells were subjected using a reporter assay. **d-g)** Immunoblot of USP25 in the RKO cells stably expressing control and USP25 shRNA. Survival assays for control and USP25 knockdown RKO cells for CCK8 assay in response to the indicatied concentration of IR (0, 1,1.5,2,2.5,3Gy), cisplatin(0,0.5,1,3µM) or 5-Fu (0, 0.1,0.5,1,3µM) for 72h. **h-i)** Immunoblot of USP25 in the SW620 cells stably expressing control and USP25 shRNA. Survival assays for control and USP25 knockdown SW620 cells for CCK8 assay in response to the indicatied concentration of 5-Fu (0, 0.1,0.5,1,3µM) for 72h. **j-k)**  Immunoblot of TRIM25 in HCT116 cells stably expressing control or TRIM25 shRNA. Survival assays for control or TRIM25 knockdown HCT116 cells for CCK8 assay in response to the indicatied concentration of 5-Fu (0, 0.1,0.5,1,3µM) for 72h. **l-m)** Immunoblot of TRIM25 in SW480 cells stably expressing control or TRIM25 shRNA. Survival assays for control or TRIM25 knockdown SW480 cells for CCK8 assay in response to the indicatied concentration of 5-Fu (0, 0.1,0.5,1,3µM) for 72h. Statistical analysis was performed using *t*-test (a) or one-way ANOVA followed by a Turkey’s multiple comparison test (c) or two-way ANOVA followed by a Turkey’s multiple comparison test (e,f,g,i,k,m) .

**
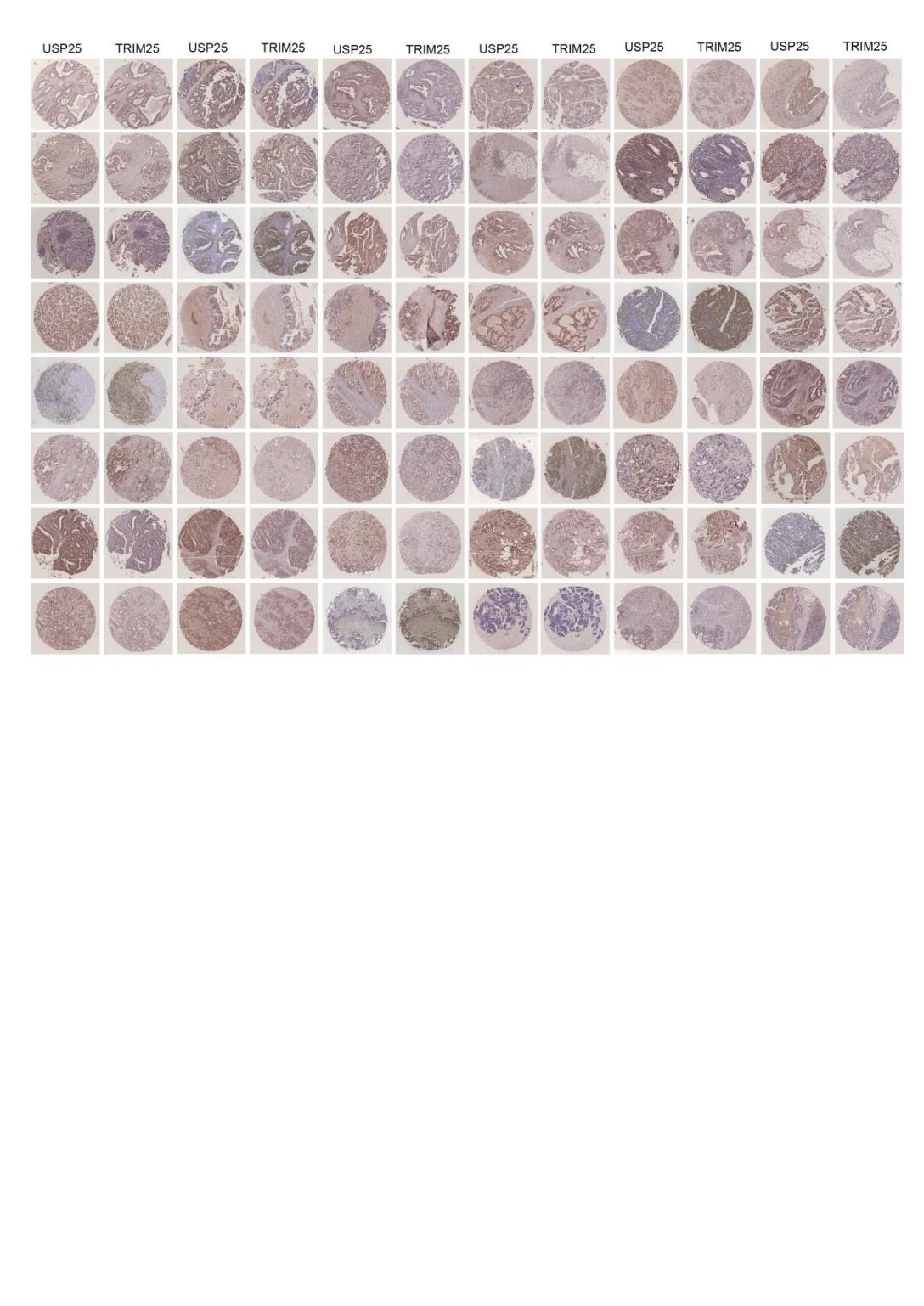
**

**Figure S6. Immunohistochemistry staining of human COAD samples，related to Figure 6.**

Tissue microarray with representative IHC images showing USP25 or TRIM25 protein expression in COAD (n=48 pairs of tumors ), respectively.

**
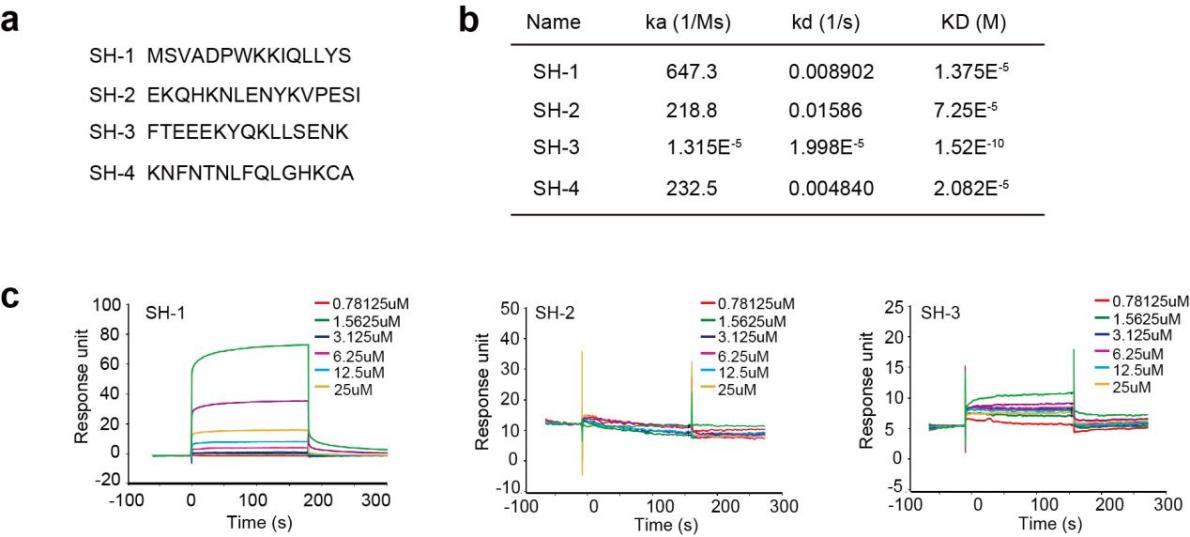
**

**Figure S7. Disturbing the USP25 and SHLD2 interaction counteracts colon cancer progression, related to Figure 7. a-c)**

The amino acid (aa) sequences of peptides covering the SHLD2-binding region from USP25 are shown. The kinetic interaction of peptides and USP25 was determined by surface plasmon resonance (SPR) analyses.
